# Profiling of Altered Metabolomic States in *Nicotiana tabacum* Cells Induced by Priming Agents

**DOI:** 10.3389/fpls.2016.01527

**Published:** 2016-10-18

**Authors:** Msizi I. Mhlongo, Paul A. Steenkamp, Lizelle A. Piater, Ntakadzeni E. Madala, Ian A. Dubery

**Affiliations:** ^1^Department of Biochemistry, University of JohannesburgAuckland Park, South Africa; ^2^Natural Products and Agroprocessing Group, Council for Scientific and Industrial Research BiosciencesPretoria, South Africa

**Keywords:** chlorogenic acids, defense responses, elicitors, hydroxycinnamates, plant activators, polyamines, priming, tyramine

## Abstract

Metabolomics has developed into a valuable tool for advancing our understanding of plant metabolism. Plant innate immune defenses can be activated and enhanced so that, subsequent to being pre-sensitized, plants are able to launch a stronger and faster defense response upon exposure to pathogenic microorganisms, a phenomenon known as priming. Here, three contrasting chemical activators, namely acibenzolar-S-methyl, azelaic acid and riboflavin, were used to induce a primed state in *Nicotiana tabacum* cells. Identified biomarkers were then compared to responses induced by three phytohormones—abscisic acid, methyljasmonate, and salicylic acid. Altered metabolomes were studied using a metabolite fingerprinting approach based on liquid chromatography and mass spectrometry. Multivariate data models indicated that these inducers cause time-dependent metabolic perturbations in the cultured cells and revealed biomarkers of which the levels are affected by these agents. A total of 34 metabolites were annotated from the mass spectral data and online databases. Venn diagrams were used to identify common biomarkers as well as those unique to a specific agent. Results implicate 20 cinnamic acid derivatives conjugated to (i) quinic acid (chlorogenic acids), (ii) tyramine, (iii) polyamines, or (iv) glucose as discriminatory biomarkers of priming in tobacco cells. Functional roles for most of these metabolites in plant defense responses could thus be proposed. Metabolites induced by the activators belong to the early phenylpropanoid pathway, which indicates that different stimuli can activate similar pathways but with different metabolite fingerprints. Possible linkages to phytohormone-dependent pathways at a metabolomic level were indicated in the case of cells treated with salicylic acid and methyljasmonate. The results contribute to a better understanding of the priming phenomenon and advance our knowledge of cinnamic acid derivatives as versatile defense metabolites.

## Introduction

Plants are constantly exposed to and attacked by pathogenic microorganisms. Plant immunity, as a multi-layered response, consists of pre-formed physical barriers and inducible immune responses. A passive resistance protection system that involves an array of structural barriers and pre-formed antimicrobial metabolites prevents or attenuates invasion by potential attackers (Pieterse et al., 2009; Denancé et al., 2013). In addition, plants employ a number of chemical defenses consisting of secondary metabolites that act as signaling molecules, antimicrobials (i.e., phytoanticipins and phytoalexins), herbivore feeding deterrents and cell wall strengthening precursors. However, certain plant pathogens can manipulate or overcome these chemical defenses to establish successful infections that have a negative impact on plant growth and subsequent agricultural yield. To overcome this, the agricultural industry has relied on the use of agrochemicals such as pesticides and fungicides (Denancé et al., 2013). However, continuous application of such chemicals have negative impacts on the environment. Hence, there is a need for new environmentally-friendly ways to combat these diseases and pests by boosting natural innate immune systems in plants. The enhancement of immunity, i.e., the induction of a physiological state that allows a plant to deploy a more rapid and stronger defense response (Balmer et al., 2013; Gamir et al., 2014; Tugizimana et al., 2014), would therefore be advantageous.

Plant “priming” is such a process of plant immune augmentation. Direct immune enhancement (*cis* priming) involves the use of a disease causing agent to induce resistance against it, while indirect immune enhancement (*trans* priming) can be obtained through treatment with biological—or chemical elicitors such that the plant becomes tolerant or resistant to a number of pathogens (Hilker et al., 2015). Recent developments in—omic technologies have provided some insights into the mechanism(s) of priming (Conrath, 2011; Walters et al., 2013; Balmer et al., 2015). These studies have indicated that priming is a result of altered metabolism, activation of dormant mitogen-activated protein kinases, chromatin modification, altered gene expression (Conrath, 2011; Greco et al., 2012) as well as secondary metabolite production (Dempsey and Klessig, 2012; Shah and Zeier, 2013).

It can be expected that, at a metabolomic level, priming would be controlled by various metabolic pathways stimulated by different endogenous—and external environmental factors. Priming mechanisms might thus vary from species to species with different outcomes for different priming agents (Walters et al., 2013; Gamir et al., 2014). Insights into priming mechanisms have been facilitated by recent advances in analytical techniques that allow the identification and quantification of priming-mediating secondary metabolites such as pipecolic acid (Návarová et al., 2012) and azelaic acid (Jung et al., 2009; Aliferis and Jabaji, 2012). Other resistance-related metabolites are terpenoids, benzoates, flavonoids, alkaloids, fatty acids, and phenylpropanoids (Bollina et al., 2010).

In order to investigate to what extent primed metabolomes overlap, a metabolomic profiling approach was used to analyze extracts obtained from *Nicotiana tabacum* cell suspensions treated with three chemical-derived elicitors with different modes of action: acibenzolar-S-methyl (ASM—Buonaurio et al., 2002), riboflavin (RIB—Liu et al., 2010), and azeleic acid (AZA—Jung et al., 2009). Ultra-high performance liquid chromatography coupled to tandem mass spectrometry (UHPLC-MS/MS) in combination with multivariate data statistical tools were used to uncover the effect of these treatments, and to further identify metabolites (biomarkers) perturbed by the different treatments. The data was then compared to that obtained from phytohormone-treated [salicylic acid (SA), methyljasmonate (MJ), and abscisic acid (ABA)] cells (Mhlongo et al., 2016) to investigate possible linkages to phytohormone-dependent pathways at a metabolomic level.

## Materials and methods

### Cell culture and -elicitation

*N. tabacum* cv. Samsun cells were used and cultivated as described by Sanabria and Dubery (2006). In short, cell cultures were grown at 25°C in Murashige and Skoog (MS) medium containing 0.25 mg/L 2,4-dichlorophenoxyacetic acid and 0.25 mg/L kinetin (pH 5.8), whilst continuously shaking at 120 rpm. Cells were subcultured into fresh medium every 7 days. Three days after subculturing, cells were treated with three chemical elicitors (ASM, AZA, and RIB) and three phytohormones (ABA, SA, and MJ). For chemical treatment the concentrations were in accordance with previous published conditions, as follow: 0.1 mM AZA (Jung et al., 2009), 1.0 mM RIB (Liu et al., 2010), and 0.3 mM ASM (Buonaurio et al., 2002). The phytohormone concentrations used were 0.1 mM ABA (Pospíšilová et al., 2005), 0.2 mM MJ (Shohael et al., 2007), and 0.3 mM SA (Mahalakshmi et al., 2013). All chemicals were dissolved in MS basal medium, except ASM which was dissolved in acetone. Suspensions were continuously agitated at 80 rpm and 25°C for 24 h. The control cells received no treatment and were incubated for 24 h. Each experiment was repeated three times to generate data corresponding to three biological replicates required for metabolomic data analysis.

### Metabolite extraction

The experimental design included three biological replicates each with three technical repeats to maximize data reproducibility and statistical reliability. Cells were harvested every 6 h after elicitation over a 24 h incubation period. Vacuum filtration was use to harvest the cells that were then washed with 20 mL MS basal medium. Two grams of cells were homogenized in 20 mL HPLC-grade methanol (1:10 m/v) using a sonicator (Bandelin Sonopuls, Germany) set at 55% power for 15 s, repeated twice. To pellet the cell debris, the homogenates were centrifuged at 5000 rpm for 20 min and the supernatants transferred into new sterile 50 mL Falcon tubes. The 20 ml supernatants were evaporated to ~1 mL at 55°C using a rotary evaporator. The ~1 mL supernatants were transferred into 2 mL sterile Eppendorf tubes and evaporated to dryness in a heating block set at 55°C overnight. The remaining pellets were re-dissolved in 400 μL 50% (v/v) HPLC-grade methanol in milliQ water and filtered through a 0.22 μm nylon filter into glass vials fitted with 500 μL inserts. The filtered extracts were stored at −20°C until being analyzed.

### Sample analysis using ultra-high performance liquid chromatography coupled to high definition mass spectrometry (UHPLC-HDMS)

One microliter of the methanol extracts was analyzed on an UHPLC-high definition quadrupole time-of-flight high-definition MS instrument (UHPLC-qTOF SYNAPT G1 HD-MS system, Waters Corporation, Manchester, UK). Compounds were separated on a T3 Acquity column (1.7 μm, 2.2 × 150 mm; Waters Corporation, Manchester, UK) using 0.1% formic acid in water (solvent A) and UHPLC-grade acetonitrile containing 0.1% formic acid (solvent B). The column temperature was maintained at 40°C. A stepwise gradient method at constant rate of 0.4 mL/min was used to elute the column with the following conditions: 5% B over 0.0–2.0 min, 5–12% B over 2.0–2.10 min, 12–65% B over 2.10–10.50 min, 65–95% B over 10.50–11.00 min, held constant at 95% B over 11.00–12.00 min, and returning from 95 to 5% B over 12.00–13.00 min. The column was washed with 5% B over 13.00–15.00 min to return to the initial conditions. The photo diode array (PDA) detector scanning range was from 200 to 500 nm with 1.2 nm resolution and a sampling rate of 20 points/s. The MS detector was set to collect both negative (ESI^−^) and positive (ESI^+^) ions. However, positive data had ionization instability and upon data analysis did not produced well-clustered PCAs. Hence, only negative ionization data is presented here. The conditions were as follows: capillary voltage 2.5 kV, sample cone voltage: 30 V, microchannel plate (MCP) detector voltage: 1600 V, source temperature: 120°C, desolvation temperature: 400°C, cone gas flow: 50 L/h, desolvation gas flow: 800 L/h, *m/z* range: 100–1000, scan time: 0.15 s, interscan delay: 0.02 s, mode: centroid, lockmass: leucine enkephalin (556.3 ng/μL), lockmass flow rate: 0.4 mL/min, mass accuracy window: 0.5 mDa. Each sample originating from the three biological replicates was analyzed in triplicate. For data acquisition pooled samples were used for quality control checks. Sample acquisition was randomized and the QC sample analyzed every 10 injections to monitor and correct changes in the instrument response.

To assist with the downstream annotation and identification of the biomarkers associated with these treatments, the MS experiment file was setup to perform unfragmented as well as five fragmenting experiments (MS^E^) simultaneously. Ion fragmentation was performed by increasing the in-source collision energy (3–30 eV; Madala et al., 2014; Ncube et al., 2014).

### Multivariate data analyses for biomarker identification

To identify biomarkers associated with the treatments, the UHPLC-MS data was analyzed with multivariate statistical tools available in the SIMCA (Soft independent modeling of class analogy) version 13.0 software (Umetrics, Umea, Sweden). Both principal component analysis (PCA) and orthogonal projection to latent structures discriminant analysis (OPLS-DA) are widely used, however, each has its own limitations. Hence, XCMS online (an automated, web-based metabolomics data processing software for biomarkers identification) was used to complement the SIMCA analyses. The workflow followed is summarized in Supplementary Material File 1, Figure S1.

#### MassLynx and SIMCA 13.0 data analysis

The UHPLC-MS ESI negative data was analyzed by MassLynx XS™ software (Waters Corporation, Manchester, UK) with added statistical programs for multivariate data analysis (MVDA). Primary raw data was pre-processed by MarkerLynx XS™ software (Waters, Manchester, UK) with the following parameters: Rt range of 2.5–11 min, mass range of 100–1000 Da, mass tolerance of 0.02 Da, Rt window of 0.2 min. Data was normalized to total intensity (area) using MarkerLynx™. The dataset obtained from MarkerLynx™ processing was exported to the SIMCA (Soft independent modeling of class analogy) version 13.0 software (Umetrics, Umea, Sweden) in order to perform PCA and OPLS-DA modeling. *Pareto* scaling was used for both the model types. PCA and OPLS-DA score plots were used to visualize and explain the metabolic differences in the samples. The generated models were evaluated by metabolomics diagnostics tools, namely the cumulative model variation in the matrix X, goodness-of-fit parameter [*R*^2^X(cum)], the proportion of the variance of the response variable that is explained by the model, *R*^2^Y(cum) and predictive ability parameter *Q*^2^(cum), also known as the total variation fraction of matrix X predicted by an extracted components (Ni et al., 2008; Sadeghi-bazargani et al., 2011). The OPLS-DA was further validated using CV-ANOVA (analysis of variance testing of cross-validation predictive residuals), where a *p* < 0.05 is an indication of a good model (Eriksson et al., 2008). Metabolites which were positively correlated to the treatments were highlighted by the PCA loading plots and OPLS-DA S-plots, where in the latter only significant metabolites with the correlation [*P(corr)*] of ≥ 0.6 and covariance of (*p*1) ≥ 0.5 were chosen for metabolite identification using the *m/z* to generate elemental composition.

#### XCMS-online data analysis

Like OPLS-DA, XCMS uses two pre-defined conditions to predict and analyze metabolome changes in the samples under study and was used to complement SIMCA 13.0 analyses since it offers additional features such as fold change and associated *p*-values. The XCMS online statistical package (https://xcmsonline.scripps.edu) is an automated web-based metabolomics data processing software that identifies biomarker features of which the relative intensity varies between sample groups for MVDA and is linked to the METLIN database (https://metlin.scripps.edu/). The software calculates both the fold change and the *p*-value of the discriminating features (biomarkers). Here, the data was processed as described (Mhlongo et al., 2014, 2016) with the following UHPLC/UHD-qTOF parameters: (i) feature detection set as centWave method, minimum peak width = 5, maximum peak width = 20, (ii) Rt correction set as Obiwarp method, Profstep = 1, alignment set as *m/z* width = 0.015, minfraction = 0.5, bw = 5, and statistics set as statistical test = Unpaired parametric *t*-test (Welch *t*-test), paired *t*-test and *post-hoc* analysis with the threshold *p* = 0.01 and fold-change = 1.5. Upon completion of the XCMS analyses, PCA score, and Cloud plots were generated. The PCA reduces data dimensionality and the Cloud plot shows both positively and negatively correlated significant features to the treatment.

### Biomarker annotation and metabolite identification

Mass fragment patterns obtained during MS analysis were used to elucidate both structural and chemical identities of analytes. MS spectral-based metabolite identification was based on (1) sufficient and accurate mass fragment information, (2) elemental composition formula calculation, (3) database search and spectral search, (4) MS^2^ to further fragment the molecule to obtain more structural information, and (5) use of authentic standards (Bateman et al., 2007; Moco et al., 2007; Brown et al., 2009).

In addition, metabolite annotation was based on the in-source collision-induced dissociation method (ISCID) previously described by our laboratory (Madala et al., 2014; Ncube et al., 2014). In short, for each positively correlated feature a single ion chromatogram was extracted and its spectral fragment pattern was compared between the different energies and published data. The mass spectrum of each extracted ion peak was used to obtain a putative empirical formula, which was used to search databases such a Dictionary of Natural Products (dnp.chemnetbase.com/) and ChemSpider (www.chemspider.com) for compound identity matches.

## Results and discussion

### Profiling of altered metabolomic states induced by priming agents

**Figure 1:**
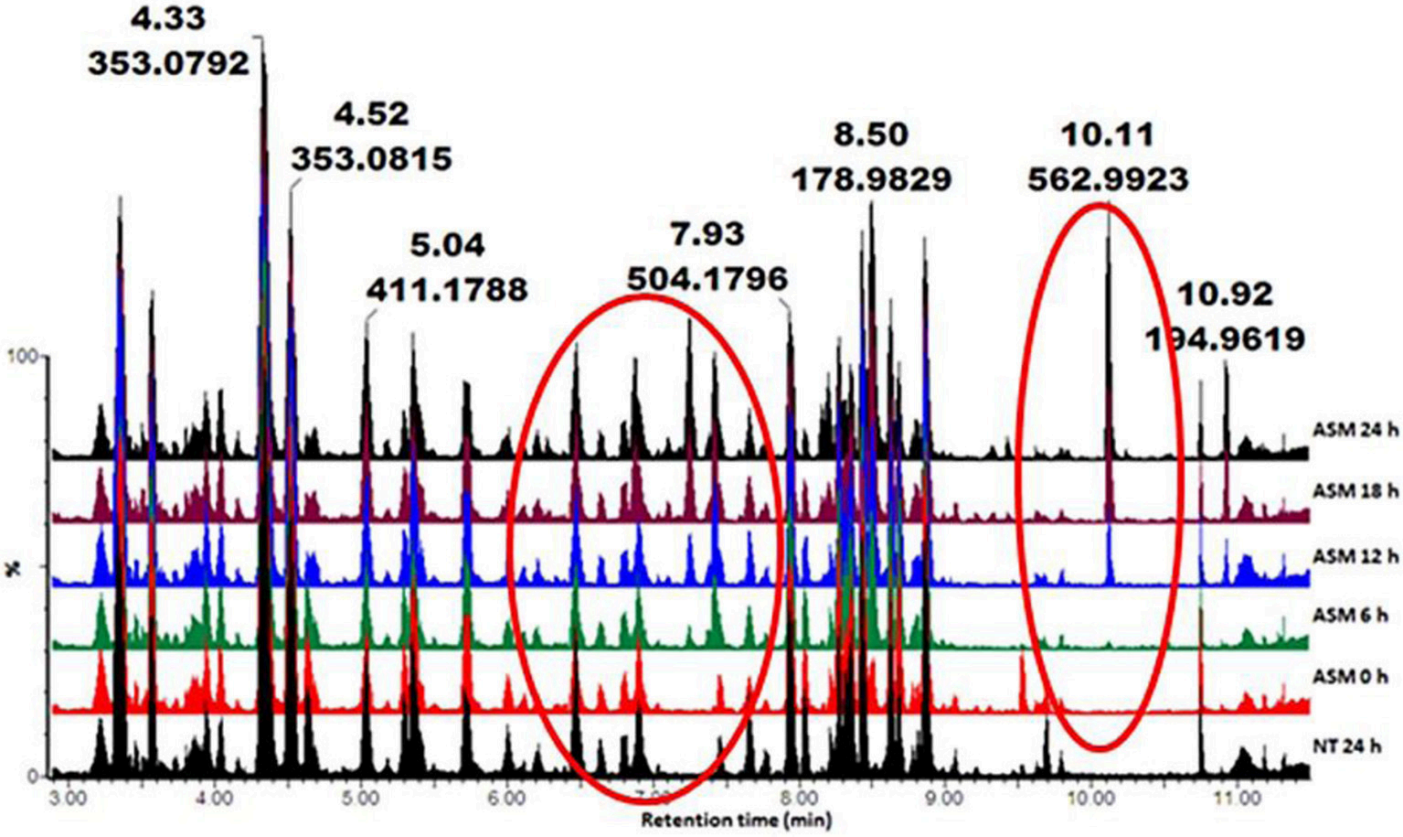
**UHPLC-MS BPI chromatograms (ESI^−^) of methanol extracts from tobacco cells treated with ASM**. The samples were extracted from cells harvested at different time intervals (0–24 h). NT 24 h is a non-treated sample incubated for 24 h. The chromatograms show some time-dependent variations (highlighted in red circles), reflecting metabolic changes over time.

Metabolic profiles of methanol-extracted metabolites from cells responding to the different inducers were obtained (Ncube et al., 2014; Tugizimana et al., 2014). Base peak intensity (BPI) chromatograms of the various extracts (e.g., Figure 1 for ASM) showed differences between treated and non-treated cells. This is an indication that these agents altered the cellular metabolism, resulting in time-dependent metabolic changes.

### Multivariate data analyses reveal metabolome differences and identifies biomarkers

#### PCA analysis

**Figure 2:**
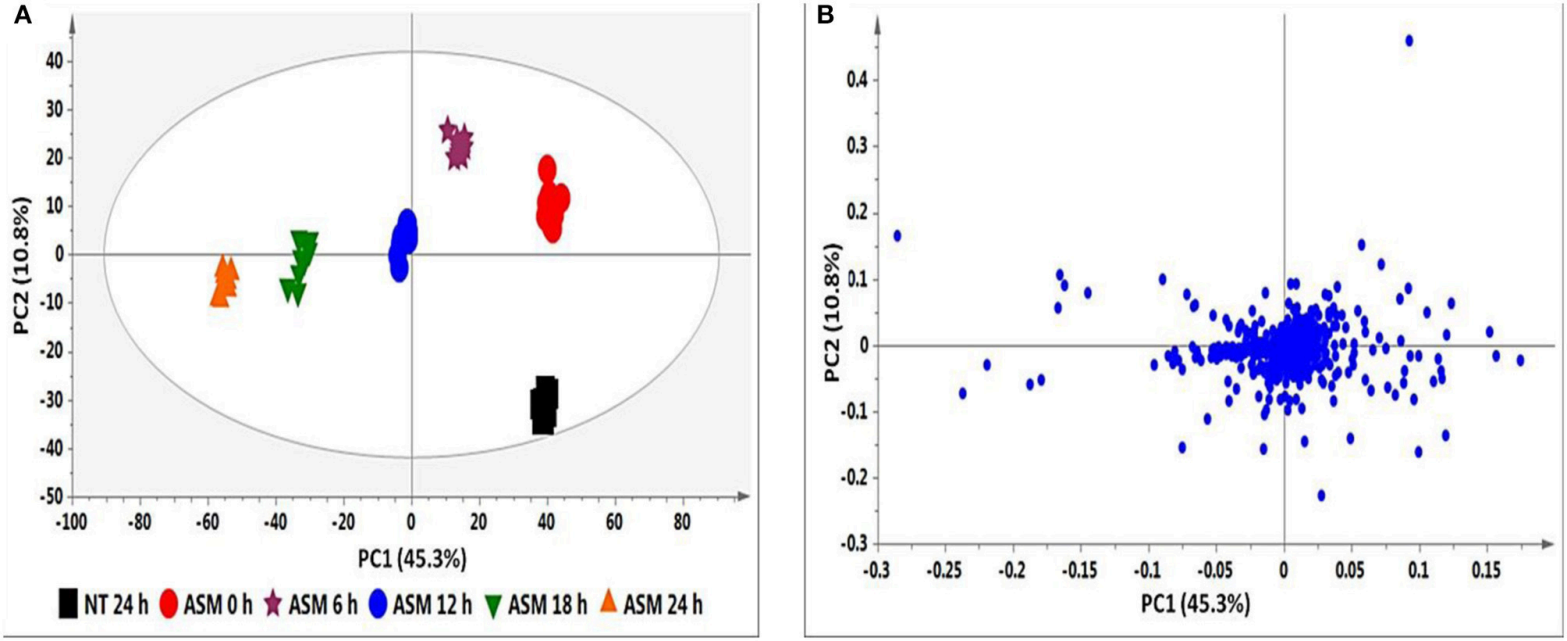
**PCA analyses of the data from ASM-treated tobacco cells. (A)** PCA score plot illustrating the clustering of samples corresponding to the different time intervals. **(B)** The corresponding loadings plot showing outliers as discriminating variables responsible for the clustering observed in **(A)**. The ellipse represents Hoteling's T2 with 95% confidence and the model calculated 4 PCs and yielded *R*^2^X = 72.1% and *Q*_(*cum*)_ = 54.7%.

The PCA score plot in Figure 2A shows a time-dependent clustering of the samples harvested at different intervals and represents the variances in the chromatograms as seen in Figure 1. The corresponding loadings plot (Figure 2B) shows features (*m/z* ions) contributing to the time-dependent clustering seen on the PCA plots (features furthest from the center). PCA aims at global visualization of similarities and differences between and within samples (explained by PC1 and PC2 respectively). However, PCA lacks predictive power and was therefore complemented with OPLS-DA.

#### OPLS-DA analysis

**Figure 3:**
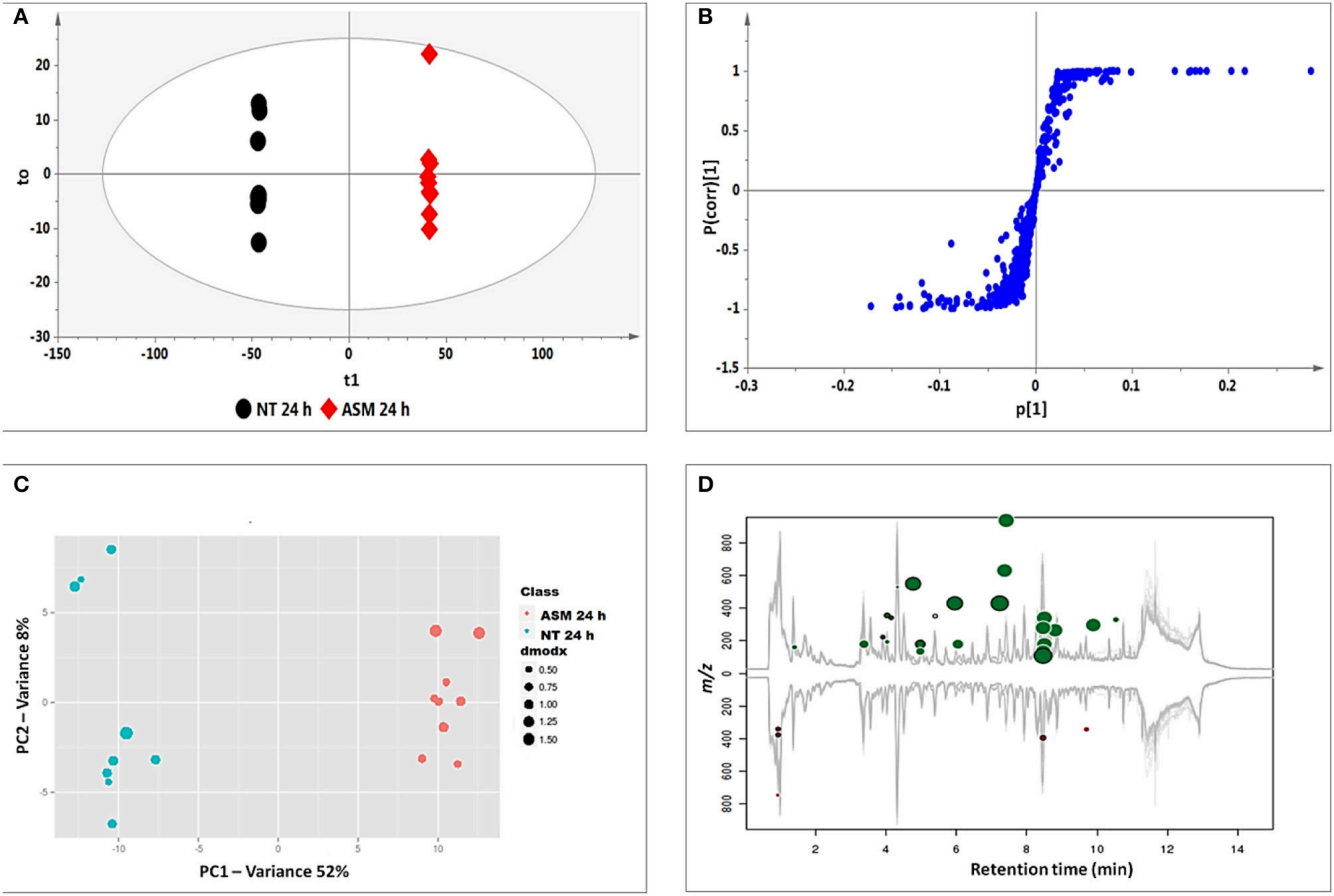
**Identification of discriminatory biomarkers: OPLS-DA (A,B) and XCMS (C,D) comparing the 24 h ASM-treated vs. 24 h non-treated tobacco cells. (A)** The OPLS-DA score plot and **(B)** the corresponding S-plot show the different clustering of treated and non-treated samples. The ellipse represents Hoteling's T2 with 95% confidence and the model calculated PCs and yielded *R*^2^X = 74.4%, *R*^2^Y = 99.9%, and *Q*_(*cum*)_ = 99.8%. Model validation by CV-ANOVA showed high model significance with *p* = 2.01 × 10^−17^. **(C)** The XCMA PCA score plot of treated vs. non-treated cells processed by using unit variance scaling centered based on features intensity. **(D)** The Cloud plot of treated vs. non-treated cells showing data set of 35 features with *p* ≤ 0.01 and fold change ≥1.5.

OPLS-DA models were calculated using two pre-defined conditions (24 h non-treated and 24 h treated) to extract differences in the samples under investigation so as to assist feature identification responsible for the observed dissimilarities (Wiklund et al., 2008; Tugizimana et al., 2013). The OPLS-DA score plots (Figure 3A) show a clear separation of the treated sample from the non-treated control. The matching S-plot (Figure 3B) was used to identify features positively correlated to the treatments. The endogenous metabolites are summarized in Table 1 and the structures presented in Figure 6, while the phytohormone metabolites and derivatives are presented in Table 2 and Figure 7.

**Table 1:** **UHPLC-MS diagnostic ions (ESI-negative mode) used for the identification of biomarkers in ASM-, AZA-, and RIB-treated tobacco cells**.

**Rt**	***m/z***	**Compound name**	**Diagnostic *m/z* fragment ions**	**ASM**	**AZA**	**RIB**	**MJ**	**SA**	**ABA**
3.44	411.172	Caffeoylputrescine glycoside **(1)**	321.14, 249.12, 179.12, 135.04	•	•		•	•	
3.59	353.086	3-Caffeoylquinic acid (2)^*^	191.05, 179.05, 135.04	•				•	
4.05	355.092	Feruloylglycoside I **(3)**	193.04	•			•		
4.38	353.085	*trans*-5-Caffeoylquinic acid (4)^*^	191.05, 135.04	•		•	•	•	
4.50	353.086	4-Caffeoylquinic acid (5)^*^	191.05, 179.03, 173.04, 135.04					•	
5.33	355.091	Feruloylglycoside II **(3)**	193.05			•			•
5.47	353.087	*cis*-5-Caffeoylquinic acid (6)^*^	191.05, 135.04	•	•	•	•		
5.77	337.194	5-*p*-Coumaroylquinic acid **(7)**	191.03			•		•	
6.05	335.071	Caffeoylshikimic acid **(8)**	179.03, 135.04			•	•		•
7.24	429.056	Caffeoylputrescine conjugate **(9)**	249.08, 179.00, 135.08	•					
7.38	630.116	*tri*-Caffeoylspermidine **(10)**	468.10	•					
7.65	474.181	Feruloyltyramine glycoside I **(11A/B)**	312.14, 178.07	•	•		•		
7.79	444.162	Coumaroyltyramine glycoside II **(12A/B)**	282.04			•			
7.95	504.183	Feruloyl-3-methoxytyramine glycoside I **(13A/B)**	342.13, 178.04				•		
8.28	474.172	Feruloyltyramine glycosides II **(11A/B)**	312.11, 178.05			•			
8.30	504.182	Feruloyl-3-methoxytyramine glycoside II **(13A/B)**	312.14, 178.07		•			•	
8.36	515.161	3,4-*di*Caffeoylquinic acid (14)^*^	535.06, 191.05, 179.03, 173.05, 135.04				•		
8.64	546.194	Feruloyl-3-methoxytyramine conjugate I **(15A/B)**	342.13, 178.10		•	•	•		
8.73	515.120	4,5-*di*Caffeoylquinic acid (16)^*^	353.03, 191.05, 179.03, 173.04, 135.04					•	
8.86	546.196	Feruloyl-3-methoxytyramine conjugate II **(15A/B)**	342.13, 178.10			•	•		

*The common and unique biomarkers in response to the priming agents are shown and compared to the phytohormones salicylic acid (SA), methyljasmonate (MJ), and abscisic acid (ABA) associated with defense responses*.

* *Authentic standards were used to validate the identification*.

**Table 2:** **UHPLC-MS diagnostic ions (ESI-negative mode) used for the identification of biomarkers in tobacco cells originating from the metabolism of azelaic acid (AZA), salicylic acid (SA), methyljasmonate (MJ), and abscisic acid (ABA)**.

**Rt**	***m/z***	**Compound name**	**Diagnostic *m/z* fragment ions**	**ASM**	**AZA**	**RIB**	**MJ**	**SA**	**ABA**
4.04	299.071	Salicylic acid glycoside **(17A/B)**	137.02					•	
4.15	313.033	Methylsalicylic acid glycoside **(18)**	151.05					•	
5.83	547.182	Azelaic acid glycoside conjugate I **(19)**	511.20, 349.14, 187.09		•				
6.30	281.134	Dihydrophaseic acid II **(20)**	231.17						•
7.02	649.230	Azelaic acid glycoside conjugate II **(21)**	349.14, 302.19, 187.10		•				
7.44	511.196	Azelaic acid *di*-glycoside **(22)**	437.16, 349.15, 187.08		•				
7.51	349.121	Azelaic acid glycoside **(23)**	187.08		•				
8.28	471.185	Abscisic acid conjugate I **(24)**	365.09, 263.12, 219.12, 153.06						•
8.33	425.180	Abscisic acid glycoside **(25)**	263.12, 219.14, 153.09						•
8.51	279.120	9′-Hydroxy-abscisic acid **(26)**	205.11						•
9.02	467.186	Abscisic acid conjugate II **(27)**	263.11, 219.11, 153.09						•
9.12	371.166	Jasmonic acid glycoside **(28)**	209.11				•		
10.39	311.166	Jasmonic acid conjugate **(29)**	209.14				•		

#### XCMS analysis

XCMS analysis was used to further investigate the variations between the two pre-defined conditions (24 h non-treated and 24 h treated extracts for all inducers). The XCMS-generated PCA score plot for ASM (Figure 3C) shows the same distinct separation as seen on the OPLS-DA-derived score plot (Figure 3A), indicating that the chosen time points differ metabolically. The Cloud plot (Figure 3D) shows the intensity of both positively (upper plot in green) and negatively (lower plot in red) correlated features to the treatment. The fold change corresponds to the bubble size (the larger the bubble, the larger the fold-change; Patti et al., 2013; Gowda et al., 2014). The *p*-value for statistical significance is represented by the intensity of the feature's color where those with low *p*-values (more statistical significant) are brighter compared to those with higher *p*-values (Benton et al., 2008; Bollina et al., 2010; Gowda et al., 2014). The y-coordinate of each feature corresponds to the *m/z* ratio while the x-coordinate is the Rt of the compound as determined by UHPLC-MS. In addition, each of the color-coded features indicated with a black border matches compounds in the METLIN mass spectral database (https://metlin.scripps.edu).

The equivalent series of figures as presented in Figures 2A,B, 3A–D for ASM were compiled for the AZA, RIB, MJ, SA, and ABA data (not shown), with the results summarized and compared in Table 1.

### Metabolite annotation and characterization

Due to the enormous variety of secondary plant metabolites, correct metabolite annotation is difficult and time-consuming. This aspect is also dependent on the database used and is subject to false positives if based on mass alone. The scarcity of authentic, commercially available plant metabolite standards also limits the number of correctly annotated molecules. In the current study, a Q-TOF-MS with mass accuracy below 3 ppm (Hossain et al., 2010) was used to profile all metabolites. The combination of high mass accuracy (within a few parts per million of the true, calculated, monoisotopic value) and high resolution permits the unambiguous determination of an empirical formula of a mass ion. To prevent incorrect usage of the IUPAC numbering system, different factors from the chromatographic separation to MS were taken into consideration as reported in previous publications (Clifford et al., 2003, 2005). The data presented here for structures **1–29** thus represents putative identifications with assigned features at level-2 annotation (Sumner et al., 2007), again since no authentic standards were available.

**Figure 4:**
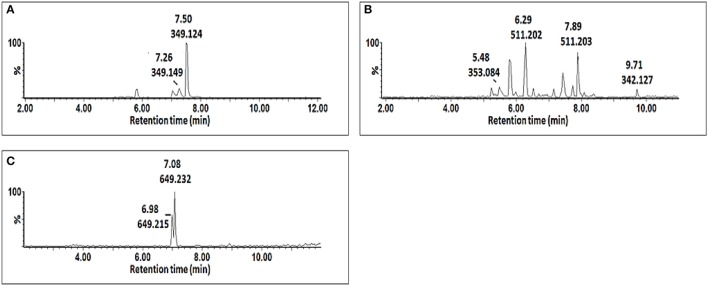
**Single ion chromatograms of UHPLC-MS showing retention times of AZA conjugates**. AZA glycoside at *m/z* 349.12 **(A)**, AZA *di*-glycoside at *m/z* 511.20 **(B)**, and AZA conjugate at *m/z* 649.23 **(C)**.

**Figure 5:**
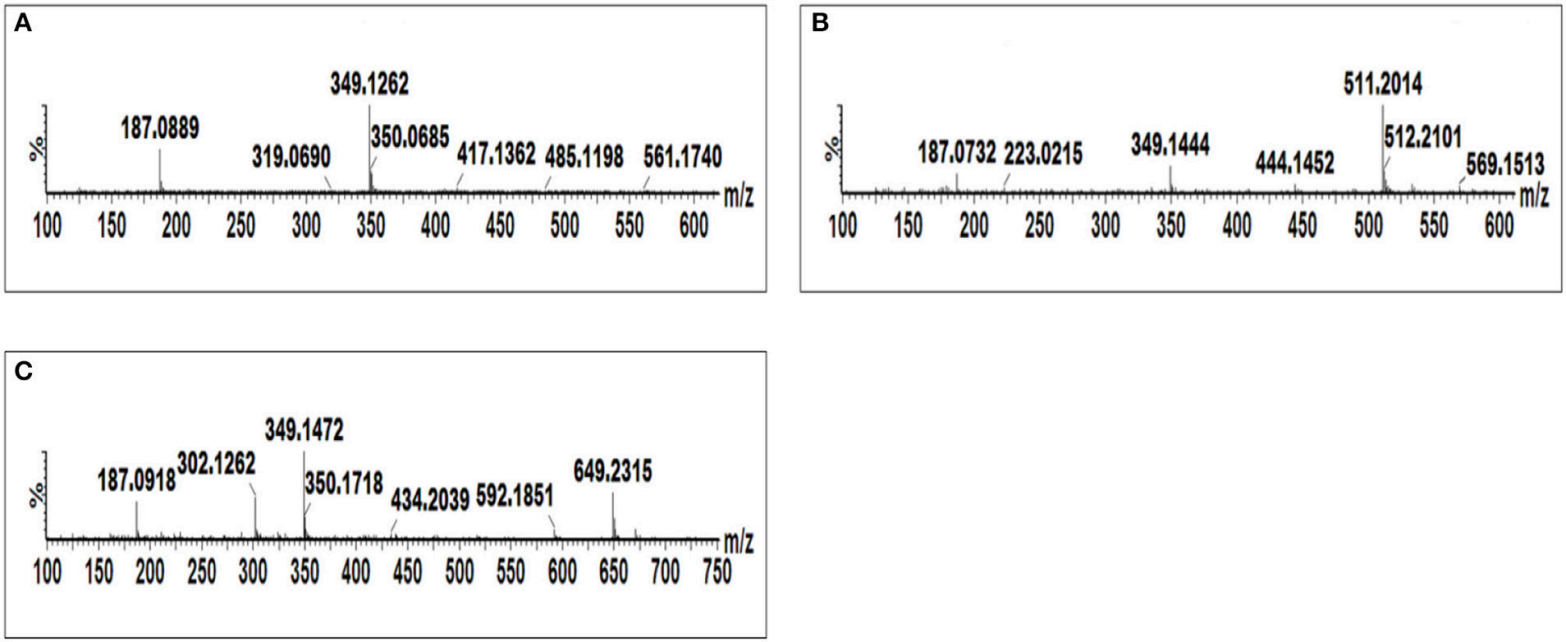
**MS spectra showing fragmentation patterns of azelaic acid derivatives, AZA glycoside (A), AZA *di*-glycoside (B), and AZA glycoside conjugate (C)**.

Procedures involving tandem MS (MS/MS) for compound identification published elsewhere (Madala et al., 2014; Ncube et al., 2014) were adopted. In short, the MS spectra were compared to published data, and online databases were also used to search generated empirical formulae. Shown below are the single ion chromatograms (Figure 4) and associated MS spectra (Figure 5) for AZA conjugates observed to accumulate in the treated cells.

AZA has a molecular weight of 188.11, generating an ESI^−^ precursor ion of 187.09. Four peaks at retention times (Rts) of 5.83, 7.02, 7.15, and 7.44 min with precursor ions of *m/z* = 547.18, 649.23, 349.12, and 511.19, respectively were found (Figure 4). Here, the MS fragmentation showed product ions at *m/z* 187.09 and corresponding to ([azelaic acid-H]^−^). The precursor at *m/z* 349.12 generated a product ion at *m/z* 187.09 ([AZA-H]^−^), indicating a loss of a glycosyl residue (C_6_H_10_O_5_) (Figure 5A). Based on this information, the molecule was annotated as AZA glycoside **(23)**. Molecule **(22)** at Rt = 7.44 min showed a precursor ion at *m/z* = 511.19 and product ions at *m/z* = 437.16 ([M-H-74 Da]^−^), 349.15 ([AZAglycoside-H]^−^) and 187.08 ([AZA-H]^−^) (Figure 5B), corresponding to the loss of two glycosyl residues. Using this information the molecule was annotated as AZA *di*-glycoside. Molecule **(21)** at Rt = 7.02 min and *m/z* = 649.230, was annotated as AZA glycoside conjugate **II**. Compound **(21)** generated MS fragments with product ions at *m/z* = 349.14 ([AZAglycoside-H]^−^) indicating a loss of an unknown residue, and one at *m/z* 187.09 ([AZA-H]^−^) representing a loss of a glycosyl residue (Figure 5C). Similarly, the fragmentation pattern of compound **(19)** (*m/z* = 547.18) corresponds to an azelaic acid glycoside conjugate with losses of two glycosyl residues. Here, the precursor at *m/z* = 547.18 generates product ions at *m/z* = 511.20 ([AZA *di*glycoside-H-36 Da]^−^) and 349.14 ([AZAglycoside-H]^−^) with the loss of another glycoside residue to generate 187.09 ([azelaic acid-H]^−^). These molecules were only identified in AZA-treated cells and the accumulation could explain metabolic attempts to sequester high AZA concentrations through transportation and storage.

**Figure 6:**
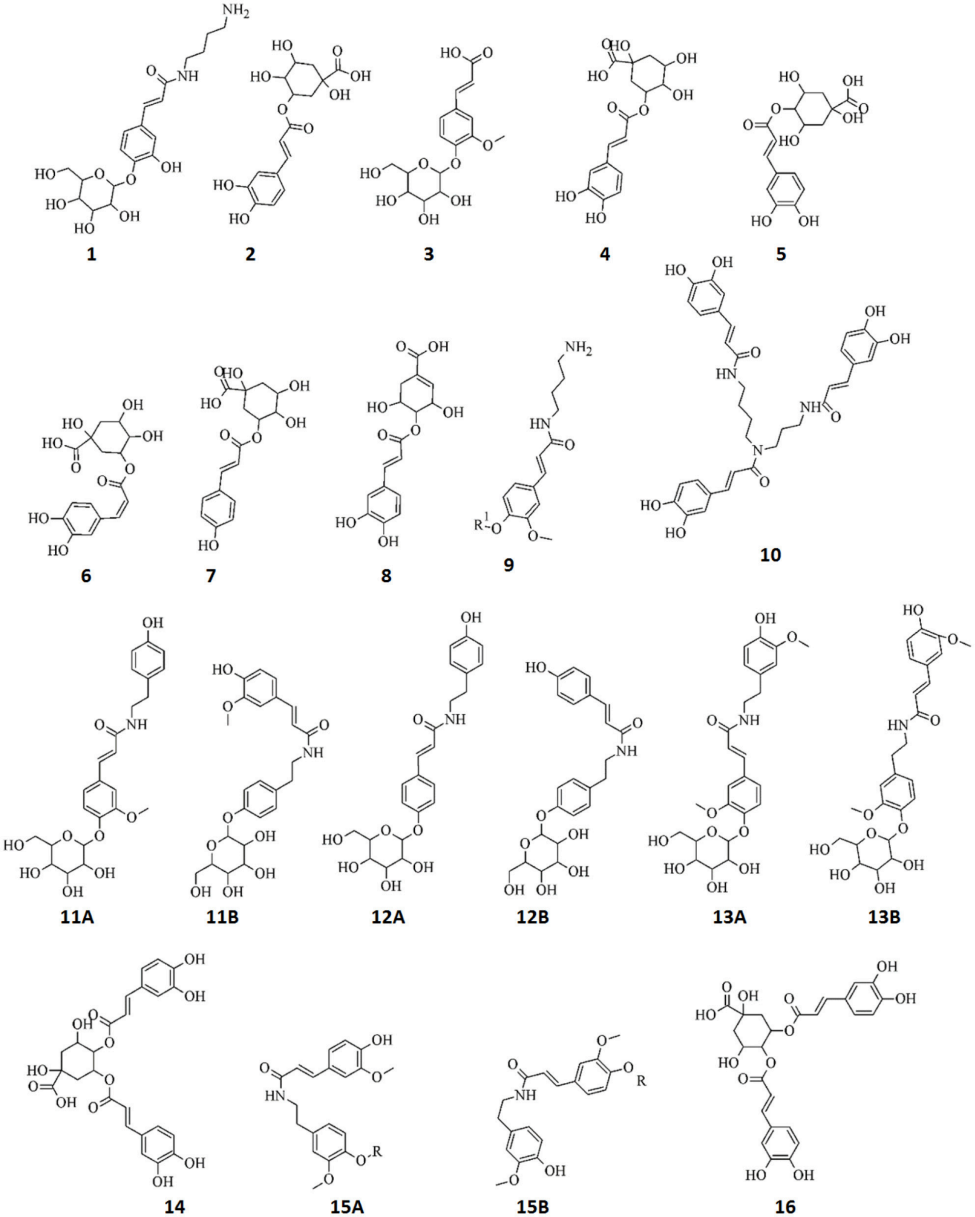
**Structures of annotated metabolites (1–16) comprising the signatory biomarkers of the priming response of tobacco cells against ASM, AZA, and RIB**. The metabolites all consist of cinnamic acid derivatives: esters, amides, and glycosides. *R* = 204 Da.

The MS data for the endogenous metabolites that were identified through MVDA analyses to be positively correlated to the ASM, AZA, and RIB treatments and their associated diagnostic peaks are presented in Supplementary Material File 2, Figures S2.1–S2.7. A total of 20 metabolites were annotated and are listed in Table 1, with structural information presented in Figure 6. All of these metabolites were found to be cinnamic acid derivatives and conjugates (esters, amides, and glycosides).

**Figure 7:**
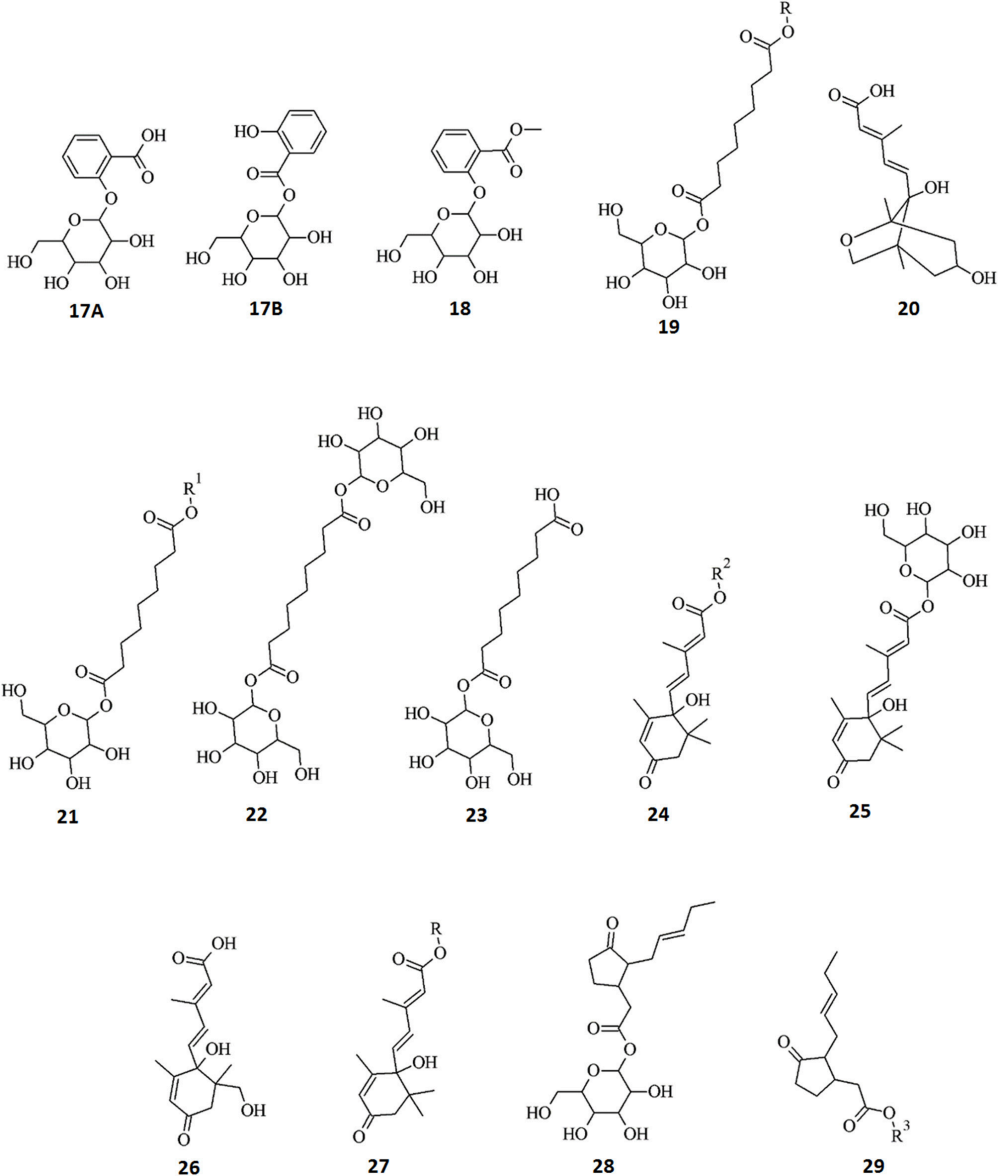
**Structures of annotated metabolites (17–29) identified as biomarkers in tobacco cells originating from the metabolism of AZA and the phytohormones ABA, MJ, and SA**. Data from hormone-treated cells indicate active metabolism thereof resulting in five ABA derivatives, two JA conjugates, and three SA derivatives. In addition, metabolism of AZA produced four derivatives: *R* = 204 Da, *R*1 = 300 Da, *R*2 = 208 Da, *R*3 = 102 Da.

Similarly, the diagnostic peaks for the derivatives of the phytohormones are shown as the corresponding single ion chromatograms and MS spectra, and are presented in Supplementary Material File 3: (ABA: Figures S3.1, S3.2), (SA: Figures S3.3, S3.4), (JA: Figures S3.5, S3.6). The annotated derivatives and catabolites are listed in Table 2, with structural information presented in Figure 7.

### Dissimilar priming agents induce similar chemical profiles in tobacco cells

The ability of plants to enter into an induced resistance state following pre-exposure to various pathogens, has facilitated the study of chemical elicitors as potential inducers of plant resistance. From these studies, a number of chemicals have been identified and some have found commercial applications as agrochemicals (Walters et al., 2005, 2013; Ishida et al., 2008; Yigit, 2011; Thakur and Sohal, 2013). Plant treatment with such compounds lead to the induction of an enhanced defensive capacity that is effective against a number of phytopathogens (Ishida et al., 2008; Walters et al., 2008, 2013). Here we used three dissimilar chemical inducers, namely ASM, AZA, and RIB, to study the priming ability at a metabolic level.

ASM is a functional analog of SA. The compound has no antimicrobial activity, but increases plant resistance by activating SAR via the SA signaling pathway (Walters et al., 2013). SAR induction by ASM against a number of bacterial, fungal and viral infections has been widely documented (Buonaurio et al., 2002; Keiko et al., 2008; Tripathi and Pappu, 2015). This compound has been commercialized as a plant health promoter under the trade names of *Bion*® or *Actigard*™ (Baysal et al., 2005).

Riboflavin is linked to a variety of flavoprotein enzyme reactions, including reduction of oxidized glutathione. It is also a potential source of singlet oxygen radicals, a reactive oxygen species, generated by plant cells responding to pathogen attack (Shetty et al., 2008). Defense induction by riboflavin is related to protein kinases and *NPR1* gene activation, but with no SA accumulation (Liu et al., 2010).

AZA functions as a long distance signaling molecule for the induction of SAR in distal tissue (Jung et al., 2009; Dempsey and Klessig, 2012; Shah and Zeier, 2013; Shah et al., 2014). Even though AZA is reported in a plant defense signaling context, at the concentration used in this study it should be regarded as a chemical inducer. There are very few studies reporting the use of AZA as priming agent and it has been reported to prime *Arabidopsis* plants to accumulate SA when challenged by *Pseudomonas syringae* (Jung et al., 2009).

### Comparison of induced metabolites in response to priming by chemical activators

**Figure 8:**
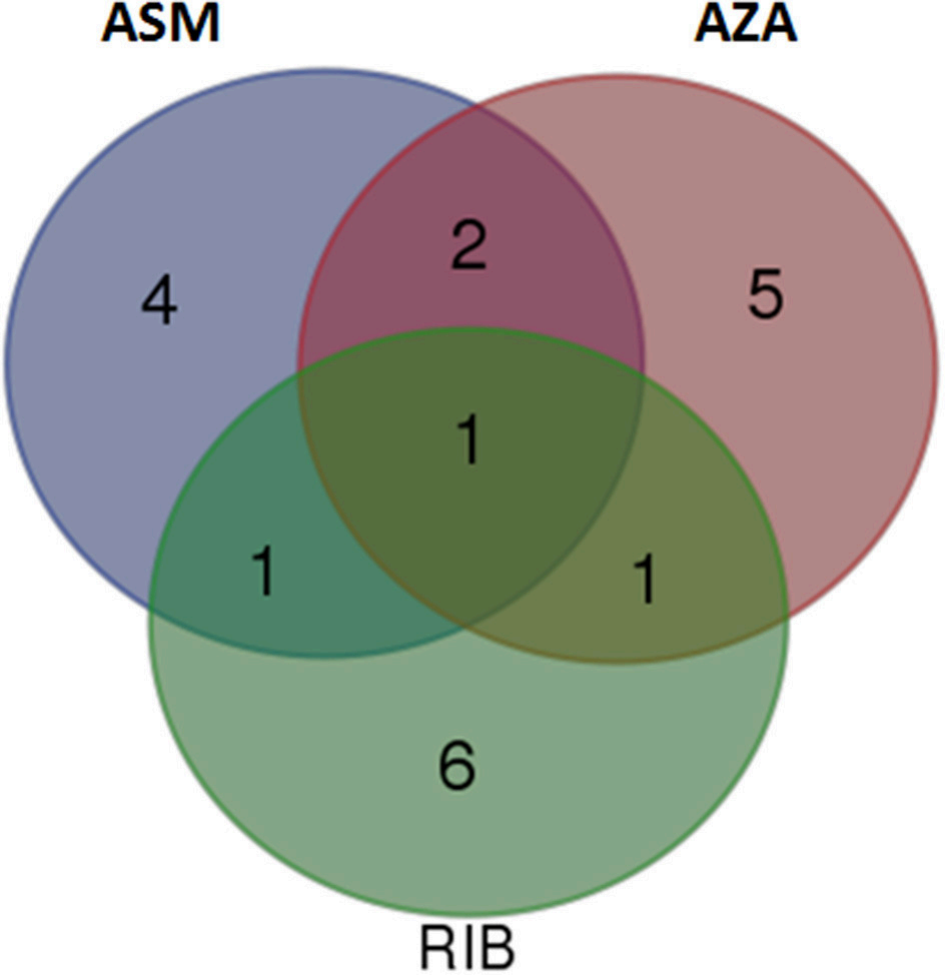
**Venn diagram of induced metabolites in tobacco cells elicited with ASM, AZA, and RIB**. The diagram shows overlapping and distinct metabolites indicated by the numbers in the intersections and circles, respectively (please refer to Tables 1, 2).

Identified biomarkers (annotated metabolites) present in extracts from cells treated with chemically diverse inducers (ASM, AZA, and RIB; listed in Tables 1, 2) were compared by means of a Venn diagram (Figure 8). Extracts from ASM- and AZA-treated cells had three metabolites in common [caffeoylputrescine glycoside **(1)**, *cis*-5-caffeoylquinic acid **(6)** and feruloyltyramine glycoside I **(11A/B)]**. AZA- and RIB-treated cells shared two metabolites [*cis*-5-caffeoylquinic acid **(6)** and feruloyl-3-methoxytryramine conjugate I **(15A/B)]**. Two chlorogenic acids [*trans*-5-caffeoylquinic acid **(4)** and *cis*-5-caffeoylquinic acid **(6)]** were common in ASM- and RIB-treated cells. Extracts from ASM-treated cells showed four unique molecules associated with the response i.e., 3-caffeoylquinic acid **(2)**, feruloylglycoside **(3)**, caffeoylputrescine conjugate **(9)**, and *tri*-caffeoylspermidine **(10)**. AZA-treated cells had five unique metabolites associated with its induced response. These included feruloyl-3-methoxytyramine glycoside II **(13A/B)** and AZA metabolites (AZA glycoside conjugate I **(19)**, AZA glycoside conjugate II **(21)**, AZA *di*-glycoside **(22)** and AZA glycoside **(23)**), associated with its own metabolism. Lastly, RIB-treated cells had six unique metabolites associated with the response: feruloylglycoside **(3)**, 5-*p*-coumaroylquinic acid **(7)**, 4-caffeoylshikimic acid **(8)**, coumaroyltyramine glycoside II **(12A/B)**, feruloyltyramine glycoside I **(11A/B)** and feruloyl-3-methoxytyramine conjugate I **(15A/B)**.

Studies have shown that induction of priming by ASM is associated with an increase in stress-responsive phenylalanine ammonia-lyase (PAL) gene expression, corresponding to increases in enzymatic activities such as PAL, chitinases and β-1-3-glucanase, and accumulation of phenolic compounds (Baysal et al., 2005; Ishida et al., 2008). PAL is the initial enzyme of the phenylpropanoid pathway leading to production of phenolic compounds such as cinnamic acid derivatives and flavonoids (Baysal et al., 2005). Pre-harvest treatment of muskmelons fruits with ASM was found to be similarly associated with the accumulation of phenolic compounds including flavonoids and lignin, thus protecting these fruits from storage diseases (Zhang et al., 2011).

As mentioned earlier, there are very few studies on AZA as a plant priming agent and, according to our knowledge, there is no induction of defense enzymes reported as yet. However, in this study it was observed that priming by AZA is associated with accumulation of cinnamic acid derivatives, suggesting PAL gene expression and an upregulated phenylpropanoid pathway. Also, the accumulation of AZA glycoside indicates that there is an AZA-UPD-glucosyl transferase that functions to sequester the excess level of AZA.

RIB-treatment of rice induces the expression of lipoxygenase (LOX), the key enzyme of the octadecanoid pathway (Taheri and Tarighi, 2010). RIB-induced resistance was suppressed in jasmonate-deficient mutants, indicating that priming by RIB is dependent on the JA signaling pathway. In addition, the expression of PAL genes was found to increase in RIB-treated plants and was associated with increased lignification. However, simultaneous application of RIB and a LOX inhibitor lead to no upregulation of the PAL gene and no lignification was observed. This implies a role for the octadecanoid pathway in the induction of the phenylpropanoid pathway, leading to lignification. This is unusual as the JA and SA pathways are known to exhibit antagonistic responses, at least in differentiated tissues (Van der Does et al., 2013). In addition, treatment of sugar beet with RIB leads to the induction of resistance against *Rhizoctonia solani* that was associated with PAL gene expression and accumulation of phenolics (Taheri and Tarighi, 2011).

Literature indicates that priming by ASM (Baysal et al., 2005; Zhang et al., 2011; Mhlongo et al., 2014) and RIB (Taheri and Tarighi, 2011) is associated with the phenylpropanoid pathway and our findings, including those of AZA, correlate with data published elsewhere. While our results clearly demonstrate that priming by these chemicals leads to phenylpropanoid pathway activation, the resulting metabolite composition of treated cells differ significantly, indicating that the underlying mechanisms leading to priming-associated metabolite production is dependent on the type of chemical inducer.

Interestingly, one unusual metabolite, *cis*-5-caffeoylquinic acid **(6)**, was found in all elicited cells, regardless the nature of the inducer. Caffeoylquinic acids are normally synthesized as the *trans* isomers (Mhlongo et al., 2014, 2015). Cinnamic acid derivatives exhibit extensive structural complexity, mainly due to positional—and geometrical (*cis* vs. *trans*) isomerism and conjugation. In this regard, we recently showed and argued the involvement of both regional and geometrical isomerism in chlorogenic acids to be a strategy deployed by plants to maximize the defensive metabolites through isomerism (Mhlongo et al., 2016; Ramabulana et al., 2016).

### Comparison of induced metabolites in response to priming by chemical activators and phytohormones

Phytohormones such as SA, MJ, and to a lesser extent ABA, are components of defense signaling networks which regulate the launching of a specific defense pathway (Pieterse et al., 2009; Verhage et al., 2010). These hormones are known to be activators or modulators of plant defense and, in some cases, the activated pathways associated with a particular signaling molecule have been identified (Pieterse et al., 2009; Dempsey and Klessig, 2012; Denancé et al., 2013; Fu and Dong, 2013). Defense responses may be associated with specific signaling molecules or may be regulated by cross-communication between the signaling pathways (Kunkel and Brooks, 2002; Spoel and Dong, 2008; De Vleesschauwer et al., 2014). Here SA, MJ, and ABA were used to study the associated triggered metabolite responses. Metabolites specifically associated with ASM, RIB, AZA, MJ, and SA treatments are listed in Table 1.

**Figure 9:**
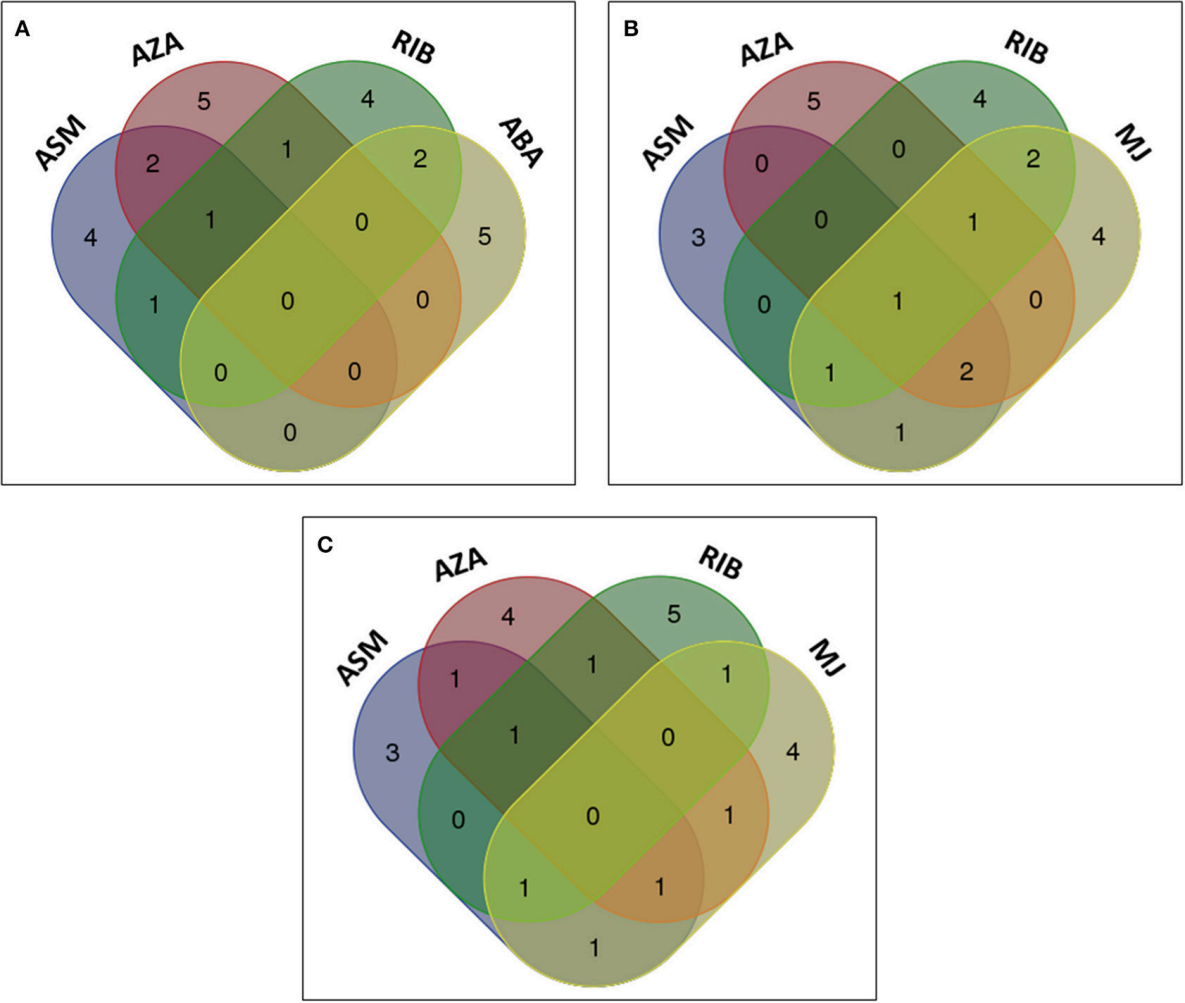
**Venn diagrams of induced metabolites in elicited tobacco cells**. Shown is the comparison of metabolites induced by chemical elicitors to those associated with **(A)** ABA, **(B)** MJ, and **(C)** SA treatment. The diagram shows overlapping and distinct metabolites indicated by the numbers in the intersections and ellipses respectively.

The metabolites from ASM-, AZA-, and RIB-treated cells were compared to those from cells treated with the phytohormones ABA, MJ, and SA to evaluate whether similar metabolites were induced, and to investigate whether the phytohormones are associated with the priming events triggered by the investigated compounds. Comparison of the chemically-induced metabolites with ABA-induced metabolites indicated that RIB- and ABA-treated cells had two metabolites in common: 4-caffeoylshikimic acid **(8)** and feruloylglycoside **(3)** (Figure 9A). Extracts from ABA-treated cells contained five unique metabolites, all associated with degradation or sequestering: *di*hydrophaseic acid **(20)**, ABA glycoside **(25)**, 9′-hydroxy-ABA **(26)** and two ABA conjugates **(24** and **27)**. ABA had a limited footprint on the cinnamic acid derivatives and no common metabolite present in extracts from all the above treatments was found.

When comparing the metabolites positively correlated to the chemical inducers with those associated with MJ, *cis*-5-caffeoylquinic acid **(6)** was also detected as a biomarker in MJ-treated cells. Two of the six RIB-treated cell metabolites were also found in MJ-treated cells (Figures 8, 9B). These metabolites were 4-caffeoylshikimic acid **(8)** and feruloyl-3-methoxytyramine conjugate **(15A/B)**. Feruloyl-3-methoxytyramine conjugate I **(15A/B)** was found to be common in AZA-, RIB-, and MJ-treated cells, caffeoylputrescine glycoside **(1)** and feruloyltyramine glycoside I **(11A/B)** were found in ASM-, AZA-, and MJ-treated cells and *trans*-5-caffeoylquinic acid **(4)** was found in ASM-, RIB-, and MJ-treated cells. Feruloylglycoside **(3)** was found in ASM- and MJ-treated cells. Extracts from ASM-treated cells had three unique associated metabolites [3-caffeoylquinic acid **(2)**, caffeoylputrescine conjugate **(9)** and *tri*-caffeoylspermidine **(10)**], and MJ-treated cells four unique metabolites: feruloyl-3-methoxytyramine glycoside **(13A/B**), 3,4-*di*caffeoylquinic acid **(14)** and two jasmonic acid (JA) metabolites; a JA glycoside **(28)** and a JA conjugate **(29)**.

Although SA was able to trigger the biosynthesis of a number of caffeoyl- and feruloyl derivatives, no single metabolite was found to be a common biomarker when compared to ASM-, AZA- and RIB- treated cells (Figure 9C). Feruloyltyramine glycoside I **(11A/B)** was found in extracts from ASM- and AZA-treated cells, feruloyl-3-methoxytryamine conjugate **(15A/B)** in AZA- and RIB-treated cells, 5-*p*-coumaroylquinic acid **(7)** in RIB- and SA-treated cells, feruloyl-3-methoxytyramine glycoside II **(13A/B)** in AZA- and SA-treated cells, 3-caffeoylquinic acid **(2)** and caffeoylputrescine glycoside **(1)** in ASM- and SA-treated cells, and *trans*-5-caffeolyquinic acid **(4)** in ASM- and RIB-treated cells. Two metabolites, namely salicylic acid glycoside (**17A/B**) and methylsalicylic acid glycoside (**18**), were only found in SA-treated cells.

### Cinnamic acid derivatives as biomarkers of priming

The relationship between the accumulation of plant phenolics as defense molecules and the priming phenomena has not been well-studied. These mechanisms are not fully understood, but could depend on a balance between biosynthesis, interconversion, conjugation, storage, and degradation (Mhlongo et al., 2014, 2016).

Chlorogenic acids (CGAs) are synthesized from hydroxylated cinnamic acids (HCAs) esterified to quinic acid [(-)-QA]. Both precursors originate from the shikimate pathway that leads to the production of HCA derivatives via PAL that catalyzes the conversion of L-phenylalanine to *trans*-cinnamic acid (Mhlongo et al., 2014, 2016). The pool of QA acts as a reservoir that can be reversibly injected into the main pathway to synthesize CGAs, e.g., via the condensation of QA with *p*-coumaroyl-CoA by hydroxycinnamoyl CoA quinate hydroxycinnamoyl transferase (Ranjeva and Boudet, 1987).

CGAs are bio-active antimicrobial molecules (Lou et al., 2011). Additional non-antimicrobial defense actions involve interfering with infection processes. In plant resistance, CGAs have been identified as phytoanticipins (Liang et al., 2006), resistance biomarkers (Leiss et al., 2011; López-Gresa et al., 2011), conferring resistance to herbivore feeding and pathogen infection (Jansen et al., 2008; Mikulic-Petkovsek et al., 2011; Ballester et al., 2013). Oxidation of CGAs by phenol oxidases yields quinones with the ability to react with amino—and sulfhydryl groups of proteins (Patil and Dimond A, 1967; Matheis and Whitaker, 1984). In this context, CGAs have been reported to inhibit enzymes used by the pathogens to infect plants including cutinase and polygalacturonase (Bostock et al., 1999; Lee and Bostock, 2007). Furthermore, CGAs were found to hinder appressorium formation (Lee and Bostock, 2007), interfering with melanin synthesis (Villarino et al., 2011) and fungal toxin production (Wojciechowska et al., 2014).

In addition to ester conjugates, HCAs were also found conjugated as amides to (i) tyramine (derived from tyrosine) and (ii) the polyamines putrescine and spermidine. The synthesis and accumulation of polyamines occurs in *N. tabacum* in response to tobacco mosaic virus-induced hypersensitive response (Torrigiani et al., 1997). In the case of barley infected with powdery mildew, agmatine conjugates were found to accumulate (Mikkelsen et al., 2015). Eight HCA amides (putrescine, tyramine, agmatine and serotonin conjugates) accumulated in wheat in response to *Fusarium graminearum* (Gunnaiah et al., 2012). Phenolic amides were also reported to accumulate in pepper leaves following bacterial lipopolysaccharide treatment (Newman et al., 2002). The molecules accumulated as a result of increased tyramine hydroxycinnamoyl transferase (THT) activity but with no increase in PAL transcripts. It was suggested that these molecules could play a role in pathogen resistance when incorporated into the cell wall (McLusky et al., 1999; Newman et al., 2002). Although the involvement of polyamines and—conjugates in plant stress responses is recognized (Alcázar et al., 2006), the precise role(s) of polyamine metabolism in these processes have remained ill-defined. Our data suggests that these amide conjugates of hydroxycinnamate derivatives fulfill a similar function as the ester conjugates to quinic acid.

The metabolic background of *N. tabacum* allows for the free HCAs as well as the ester- or amide-bound conjugates to be glycosylated, as found for several of the annotated biomarkers. Our findings are in agreement with Tugizimana et al. (2014), who reported that fungal ergosterol induced dynamic changes in *N. tabacum* cells, including caffeoylquinic acid and other HCAs conjugates. Resistance conferred by HCA derivatives seems to be a result of a dynamic balance between synthesis and degradation. We previously argued, that the reverse reactions of those leading to the HCA conjugates would release the QA, sugars, shikimic acid, polyamines (putrescine and spermidine), tyramine, and HCA derivatives (*p*-coumaric-, caffeic- and ferulic acids), which can then be incorporated into the defense arsenal of the infected plant (Mhlongo et al., 2016). Thereby we offered a plausible explanation for the accumulation of CGAs and related derivatives during priming. Sensitized plants that have been primed to accumulate high levels of HCA-ester and -amide derivatives would enable them to launch a stronger and faster defense response upon subsequent infections.

## Conclusion

Novel information at a metabolite level was generated about the primed defensive state in cultured tobacco cells in response to three non-related chemical inducers. Using a metabolic fingerprinting approach with the aid of high definition MS and multivariate statistics, biomarkers associated with a primed state induced by these agents were identified. Most of these biomarkers were early phenylpropanoid pathway intermediates and products. This indicates the importance of an activated phenylpropanoid pathway to establish a primed state in response to treatment with the inducing agents. The biomarkers associated with the chemical priming agents were compared to those triggered by defense-related phytohormones, and indicated overlap with SA- and JA-induced metabolites. The results contribute to a deeper insight into changes in secondary metabolism associated with plant priming, and implicate HCA derivatives conjugated to (i) quinic acids (as CGAs), (ii) shikimic acid, (iii) tyramine, (iv) polyamines, or (v) glucose as discriminatory biomarkers of priming in tobacco cells. Since metabolites are the end product of any physiological change and reflect dynamic variations in response to different environmental stimuli, the synthesis, accumulation, interconversion, and degradation show a true reflection of the altered physiological states induced by the priming agents. The obtained data supports a new hypothesis that the pre-emptive accumulation of these HCA derivatives and the storage as conjugates, allow the plant cells to release the HCA derivatives into the metabolic pathways leading to phytoalexin—and lignin biosynthesis upon perception of attempted pathogen attack. The results obtained in this study, therefore, contribute to an alternative view and better understanding of the functional significance of metabolite changes associated with the priming phenomenon that can be used to design a new generation of crop protection agents, able to augment existing innate immunity.

## Author contributions

Conceived and designed the research: ID, LP, NM. Performed the experiments: MM, PS. Analyzed the data: MM, NM, ID. Interpreted the data: ID. Wrote and edited the paper: MM, LP, ID.

## Funding

The research was partially funded by the South African National Research Foundation (NRF) through grant support (number 95818) to ID.

### Conflict of interest statement

The authors declare that the research was conducted in the absence of any commercial or financial relationships that could be construed as a potential conflict of interest.

## Supplementary material

Supplementary Material File 1 **Scheme illustrating the workflow for multivariate data analysis followed in the study**.

Supplementary Material File 2 **Characterization of cinnamic acid derivatives and conjugates**.

Supplementary Material File 3 **Characterization of phytohormone derivatives and catabolites**.
