# Supplementary material for: Profiling of Altered Metabolomic States in Nicotiana tabacum Cells Induced by Priming Agents
Source: Front Plant Sci. 2016 Oct 18;7:1527. doi: 10.3389/fpls.2016.01527 (PMC5068090; doi:10.3389/fpls.2016.01527)

Supplementary Material – File 1

Scheme illustrating the workflow for multivariate data analysis followed in the study.

• Profiling of altered metabolomic states in *Nicotiana tabacum* cells induced by priming agents

Msizi I. Mhlongo[1], Paul A. Steenkamp[1,2], Lizelle A. Piater[1], Ntakadzeni E. Madala[1] and Ian A. Dubery[1]*

[1]Department of Biochemistry, University of Johannesburg, Auckland Park, Johannesburg, South Africa; [2]CSIR Biosciences, Natural Products and Agroprocessing Group, Pretoria, South Africa.

*** Correspondence:**
Ian Dubery
idubery@uj.ac.za

Keywords: chlorogenic acids, defense responses, elicitors, hydroxycinnamates, tyramine, plant activators, polyamines, priming

File S1. Scheme illustrating the workflow for multivariate data analysis followed in the study. (This corresponds to section 2.4 (2.4.1 and 2.4.2) of Materials and Methods)

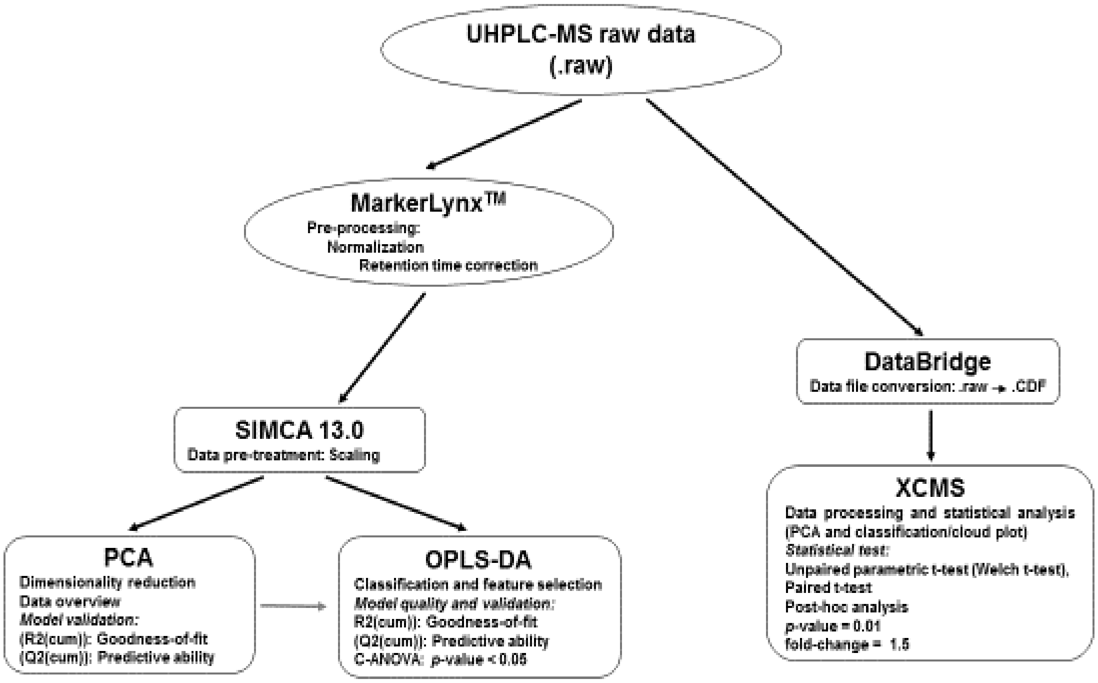

Supplement: Supplementary Material File 1 — Scheme illustrating the workflow for multivariate data analysis followed in the study. [file Image1.PDF]
