# Supplementary material for: Profiling of Altered Metabolomic States in Nicotiana tabacum Cells Induced by Priming Agents
Source: Front Plant Sci. 2016 Oct 18;7:1527. doi: 10.3389/fpls.2016.01527 (PMC5068090; doi:10.3389/fpls.2016.01527)
Supplement: Supplementary Material File 2 — Characterization of cinnamic acid derivatives and conjugates. [file Image2.PDF]

Supplementary Material – File 2

Characterization of cinnamic acid derivatives and conjugates

• Profiling of altered metabolomic states in *Nicotiana tabacum* cells induced by priming agents

Msizi I. Mhlongo[1], Paul A. Steenkamp[1,2], Lizelle A. Piater[1], Ntakadzeni E. Madala[1] and Ian A. Dubery[1]*

[1]Department of Biochemistry, University of Johannesburg, Auckland Park, Johannesburg, South Africa; [2]CSIR Biosciences, Natural Products and Agroprocessing Group, Pretoria, South Africa.

*** Correspondence:**
Ian Dubery
idubery@uj.ac.za

Keywords: chlorogenic acids, defense responses, elicitors, hydroxycinnamates, tyramine, plant activators, polyamines, priming

CHARACTERISATION OF CINNAMIC ACID DERIVATIVES AND CONJUGATES

Fig. S2.1 **Caffeoylshikimic acid (CSA) isomers.** Extracted single ion chromatograms (XIC) of UHPLC-MS/MS data showing the retention times (A) and the corresponding MS fragmentation patterns (B and C).

Fig. S2.2 ***p*-Coumaroylquinic acid (*p*-CQA)**. Extracted single ion chromatograms (XIC) of UHPLC-MS/MS data showing the retention times (A) and the corresponding MS fragmentation pattern (B).

Fig. S2.3 **Feruloylglycoside**. Extracted single ion chromatograms (XIC) of UHPLC-MS/MS data showing the retention times (A) and the corresponding MS fragmentation pattern (B).

Fig. S2.4 **Chlorogenic acids-1.** Single ion chromatograms of (**A**) *mono*-acylated chlorogenic acids and (**B**) *di*-acylated chlorogenic acids and also chlorogenic acid glycosides at Rt 3.85 and 5.04.

Fig. S2.5 **Chlorogenic acids-2**. MS spectra showing fragmentation patterns of 3-CQA (A), 4-CQA (B), *cis*/*trans*-5-CQA (C), 3,4-diCQA (D) and 4,5-diCQA (E).

Fig. S2.6 **Single ion chromatograms (XIC) of UHPLC-MS/MS showing the retention times of cinnamic acid derivatives conjugated to nitrogen containing molecules**. Caffeoylputrescine glycoside (A), *p*-coumaroyltyramine glycoside (B), feruloyltyramine glycoside (C), feruloyl-3-methoxytyramine-4-glycoside (D) and feruloyl-3-methoxytyramine conjugate (E).

Fig. S2.7 **MS spectra showing fragmentation patterns of cinnamic acid derivatives conjugated to nitrogen containing molecules:** Caffeoylputrescine glycoside (A), *p*-coumaroyltyramine glycoside (B), feruloyltyramine glycoside (C), feruloyl-3-methoxytyramine-4-glycoside (D) and feruloyl-3-methoxytyramine conjugate (E).

Cinnamic acids are phenolic compounds produced *via* the shikimate and phenylpropanoid pathways and have been reported to play an important role in plant priming against biotic and abiotic stresses. The mostly know cinnamic acids derivatives are *p*-coumaric -, caffeic - and ferulic acid which can be conjugated to quinic acid, shikimic acid (Clifford 1999, 2000), amines, amino acids and sugars (Clifford and Knight, 2004). Sinapic acids molecules conjugated to quinic acid are rarely founds in plants.

2.1 Characterization of caffeoylshikimic acids

Molecule **(8)** at Rt 6.05 min was annotated a caffeoylshikimic acid with a precursor ion at *m/z* 335.07 ($[M-H]^-$) **(Fig. S2.1A)** and with an MS spectrum showing product ions at *m/*z 178.94 $[(caffeoyl-H)^-]$ indicating a loss of a shikimic acid residue and *m/*z 135.03 $[caffeoyl-H-COO)^-]$ for a decarboxylated caffeic acid **(Fig. S2.1B)**

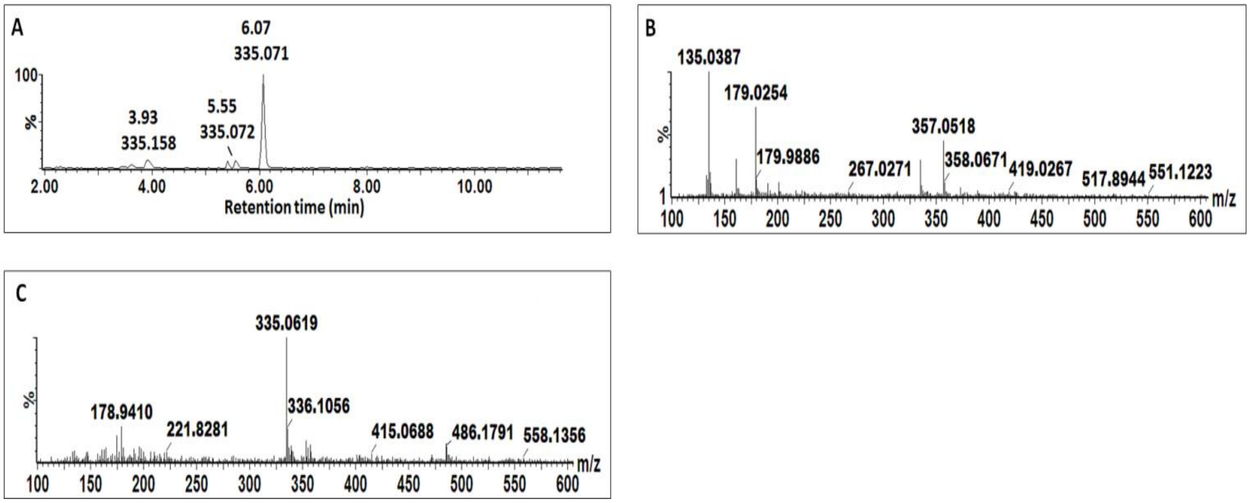

Figure S2.1 Caffeoylshikimic acid (CSA) isomers. Extracted single ion chromatograms (XIC) of UHPLC-MS/MS data showing the retention times (**A**) and the corresponding MS fragmentation patterns (**B** and **C**).

2.2 Characterization of p-coumaroylquinic acids

Molecule **(7)** with Rt 5.77 min was annotated as a 5-*p*-coumaroylquinic acids (5-*p*-CoQAs) with a precursor ion at *m/z* 337.09 ([M-H]$^-$) **(Fig. S2.2A)** and with an MS spectrum showing a product ion at *m/z* 191.05 [(quinic acid-H)$^-$] resulting from the elimination of *p*-coumaric acid **(Fig. S2.2B)**.

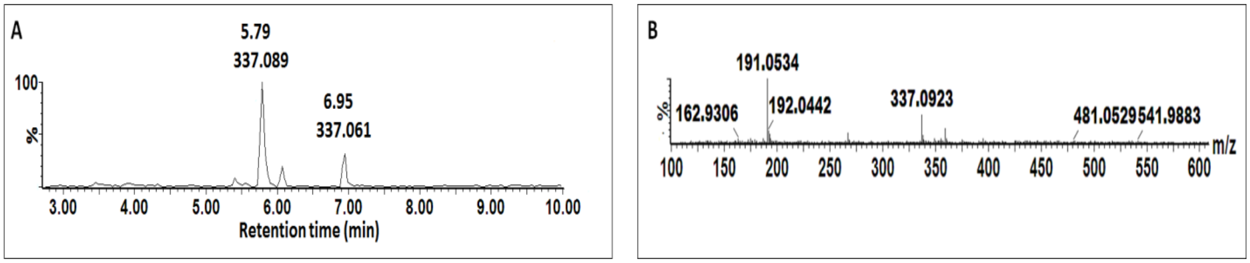

Figure S2.2 ***p*-Coumaroylquinic acid (*p*-CQA)**. Extracted single ion chromatograms (XIC) of UHPLC-MS/MS data showing the retention times (**A**) and the corresponding MS fragmentation pattern (**B**).

2.3 Characterization of feruloylglycosides

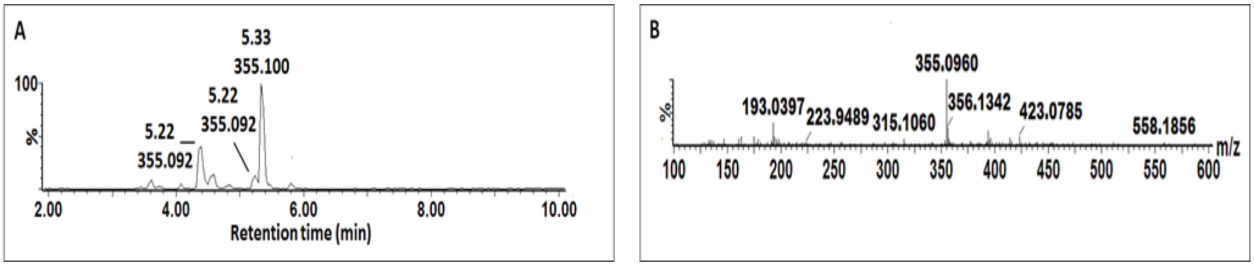

Figure S2.3 **Feruloylglycoside**. Extracted single ion chromatograms (XIC) of UHPLC-MS/MS data showing the retention times (**A**) and the corresponding MS fragmentation pattern (**B**).

Two peaks for molecule **(3)** at Rt 4.05 and 5.33 min with precursor ion at *m/z* 355.09 ([M-H]$^-$) and 355.10 ([M-H]$^-$), respectively **(Fig. S2.3A)**. Their MS spectra show product ions at *m/*z 193.04 ([ferulic acid-H]$^-$) resulting from the loss of a glucosyl residue and 175.12 ([feruloyl-H-H_2O]$^-$) **(Fig. S2.3B).** Using this information the two molecules were annotated feruloylglycoside I and II. (Jaiswal *et al*., 2014).

2.4 Characterization of mono- and di-acylated quinic acids

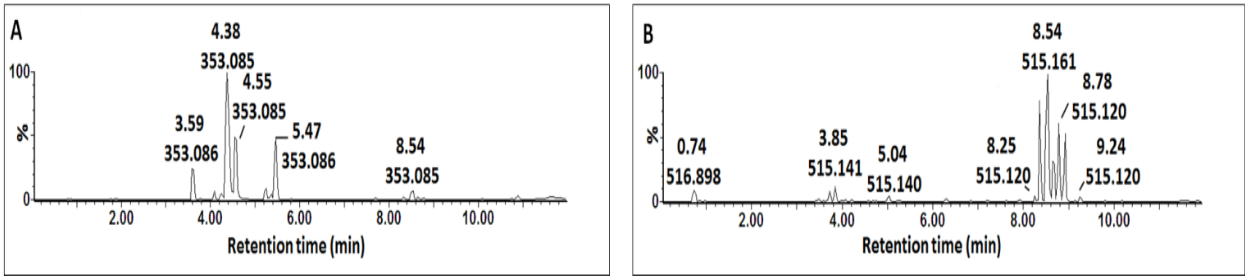

Figure S2.4 Chlorogenic acids-1. Single ion chromatograms of (**A**) *mono*-acylated chlorogenic acids and (**B**) *di*-acylated chlorogenic acids and also chlorogenic acid glycosides at Rt 3.85 and 5.04.

Caffeoylquinic acids (CQAs) are also referred to as chlorogenic acids and during MS analysis they produce common fragments that can be used to distinguish between the different *stereo*- or *regio*-isomers. These molecules have a precursor ion at *m/z* 353.08 and upon MS fragmentation yield product ions at *m/z* 191 $[(\text{quinic acid-H})^-]$, 179 $[(\text{caffeoyl-H})^-]$, 173 $[(\text{quinic acid-H-}H_2O)^-]$ and 135 $[(\text{caffeoyl-H-COO})^-]$ (He *et al.*, 2010). To be certain with regard to the positional substitutions on the quinic acid, we considered that a C-4 acylated CGA shows a product ion at *m/z* 173, whilst C-3 and C-5 acylated CGAs are distinguished by a base product ion, *m/z* 191, with the C-3 showing an additional product ion at *m/z* 179 (which is approximately 50% as intense when compared to the *m/z* 191), and C5 showing a very minimal or even undetectable *m/z* 179 **(Fig S2.5A-C)**. Using this information together with the hierarchical diagnostic fragmentation key proposed by Clifford *et al.* (2003) all *regio*-isomers were identified and correctly annotated. Here four precursor ions at *m/z* 353.08 ($[M-H]^-$) at Rts 5.39, 4.50, 5.47 and 4.38 min **(Fig. S2.4A)** were annotated as 3-CQA **(2)** (fragment ions at *m/z* 191.05, 179.12 and 135.24) **(Fig. S2.5A)**, 4-CQA **(5)** (fragment ions at *m/z* 191.04, 179.15, 173.03 and 135.04) **(Fig. S2.5B)**, *cis*-5-CQA **(6)** (fragment ions at *m/z* 191.05, and 135.14) **(Fig. S2.5C)** and *trans*-5-CQA **(4)** (fragment ions at *m/z* 191.05 and 135.14) **(Fig. S2.5C)**. Both the *cis*- and *trans*-isomers of 5-CQA produced similar spectra but could be distinguished by their different retention times; here only the *trans* isomer is indicated. Molecule **(14)** and **(16)** with precursor ion 515 ($[M-H]^-$), Rt 8.36 and 8.73 min, were annotated as 3,4-*di*-caffeoylquinic acid **(Fig. S25.B)** and 4,5-*di*-caffeoylquinic acid **(Fig. S2.5E)**, respectively.

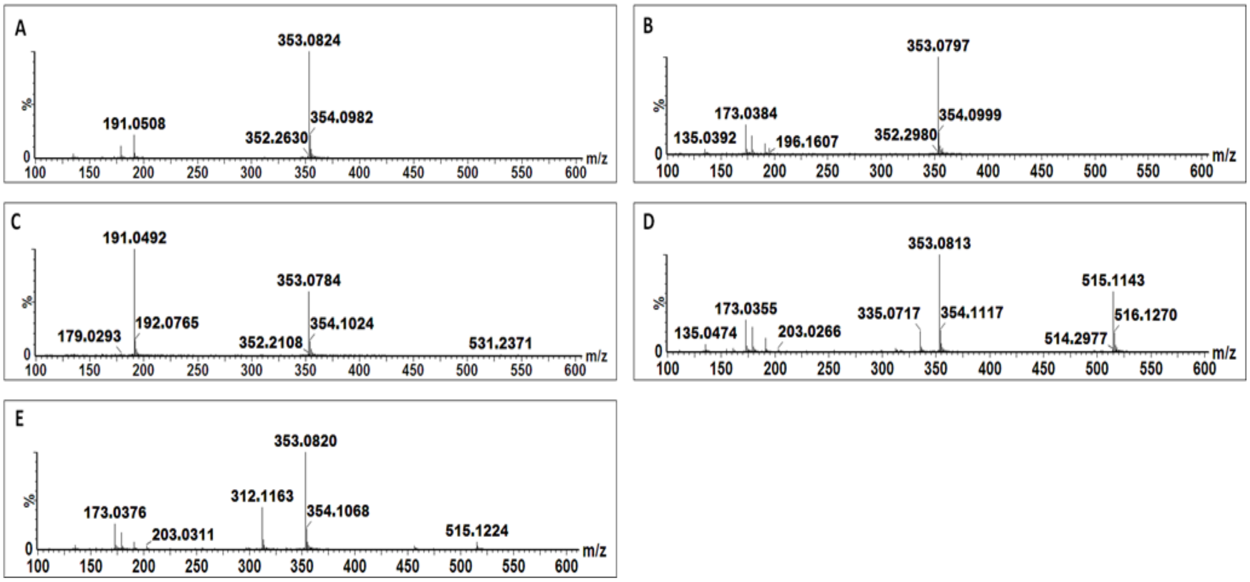

Figure S2.5 Chlorogenic acids-2. MS spectra showing fragmentation patterns of 3-CQA (**A**), 4-CQA (**B**), *cis*/*trans*-5-CQA (**C**), 3,4-diCQA (**D**) and 4,5-diCQA (**E**).

2.5 Characterization of hydroxycinnamic acid amines

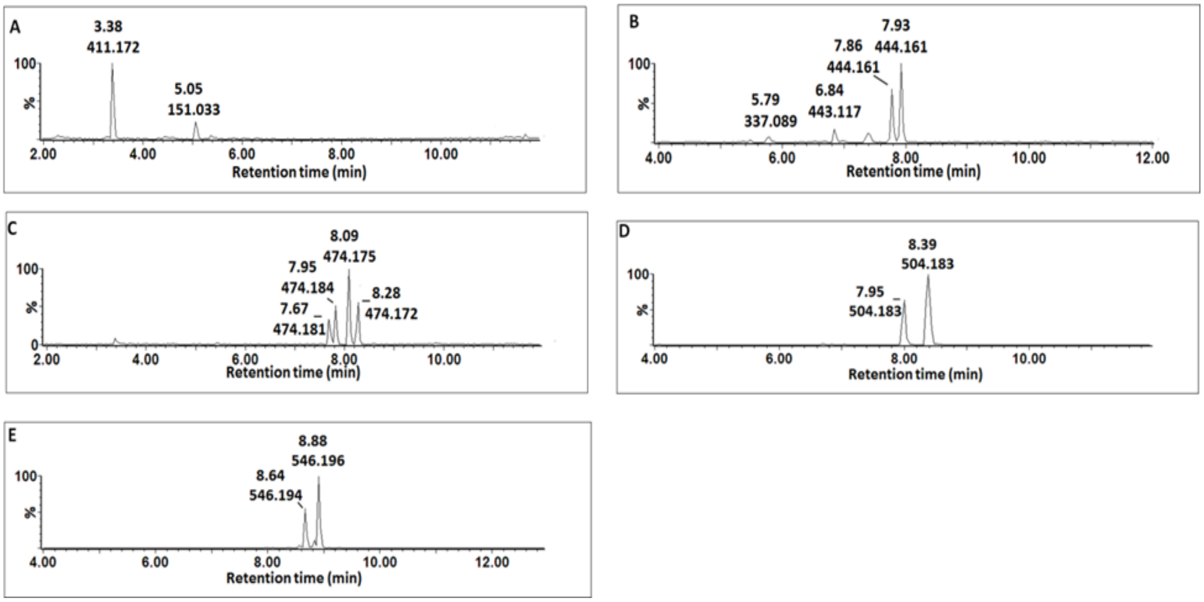

Figure S2.6 Single ion chromatograms (XIC) of UHPLC-MS/MS showing the retention times of cinnamic acid derivatives conjugated to nitrogen containing molecules. Caffeoylputrescine glycoside (**A**), *p*-coumaroyltyramine glycoside (**B**), feruloyltyramine glycoside (**C**), feruloyl-3-methoxytyramine-4-glycoside (**D**) and feruloyl-3-methoxytyramine conjugate (**E**).

Molecule **(1)** was annotated as caffeoylputrescine glycoside with a precursor ion at *m/z* 411.17 ([M-H]$^-$) Rt 3.44 min **(Fig. S2.6A),** and with an MS spectrum showing product ions at *m/z* 249.12 ([M-162 Da]$^-$) indicating a loss glucosyl residue, 178.96 ([caffeoyl-H)$^-$] and 135.05 [(caffeoyl-H-COO (C2)$^-$] **(Fig. S2.7A)**. Molecule **(20)** was annotated as caffeoylputrescine conjugate with a precursor ion at *m/z* 429.06 ([M-H]$^-$) Rt 7.24 **(Fig. S2.6B)** and with product ions at *m/z* 249.12 ([M-180 Da]$^-$), 178.96 ([caffeoyl-H]$^-$) and 135.05 ([caffeoyl-H-COO]$^-$) **(Fig. S2.7B)**. Molecule **(12A/B)** was annotated as *p*-coumaroyltyramine glycoside with a precursor ion at *m/z* 444.79 ([M-H]$^-$) Rt 7.79 (**Fig. S2.6C)** with its MS spectrum showing a product at *m/z* 282 ([coumaroyltyramin-H-162 Da]$^-$) corresponding to the loss of a glycosyl residue **(Fig. S2.7C).**

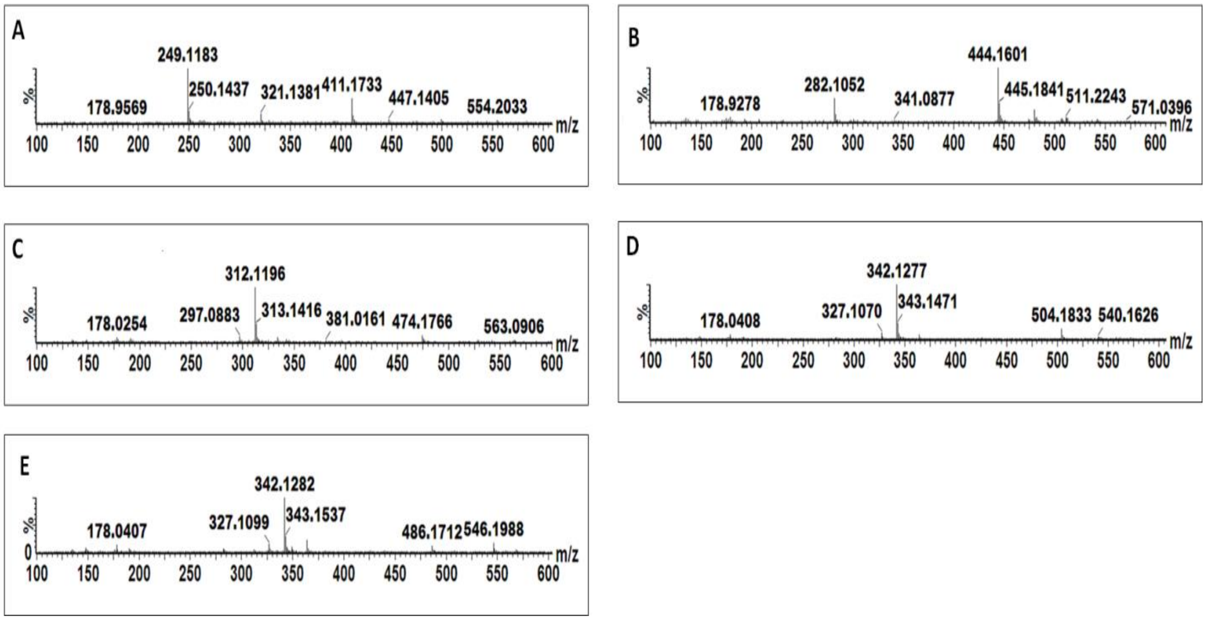

Figure S2.7 MS spectra showing fragmentation patterns of cinnamic acid derivatives conjugated to nitrogen containing molecules: Caffeoylputrescine glycoside (**A**), *p*-coumaroyltyramine glycoside (**B**), feruloyltyramine glycoside (**C**), feruloyl-3-methoxytyramine-4-glycoside (**D**) and feruloyl-3-methoxytyramine conjugate (**E**).

Molecule **(11)** was annotated as feruloyltyramine glycoside with a precursor ion at *m/z* 474.18 ($[M-H]^-$) Rt 7.65 **(Fig. S2.6D)** and it MS spectrum showed product ions at *m/*z 312.12 ($[\text{feruloyltyramine-162 Da}]^-$) as a result of glycosyl residue elimination and 178.03 ($[\text{feruloyl-H}]^-$) **(Fig. S2.7D)**. Two precursor ions at *m/*z 504.18 ($[M-H]^-$) Rt 7.95 and 8.30 min (**Fig. S2.6E)** were detected for molecule **(13A/B)** and were annotated as feruloyl-3-methoxytyramine-4-glycoside. Their MS spectra produced distinctive product ions at *m/z m/z* 342.13 ($[\text{feruloyl-3-methoxytyramine -H-162 Da}]^-$) as a result of glycosyl residue loss and 178.04 ($[\text{feruloyl-H}]^-$) after losing the feruloyl methoxy group and 3-methoxyltyramine residue **(Fig. S2.7E)**. Molecule **(15A/B)** was annotated as feruloyl-3-methoxyyramine conjugate with precursor ions at *m/z* 546.16 ($[M-H]^-$) Rt 8.64 and 8.86 min **(Fig. S2.6F)**. Their MS spectra produced product ions at *m/z* 342.13 ($[\text{M-H-203 Da}]^-$), 327.11 ($[\text{feruloyltyramine-H-}CH_3]^-$) due to the loss of a methoxy group and 178.04 ($[\text{feruloyl-H}]^-$) after losing the feruloyl methyl group and tyramine residue **(Fig. S2.7F)**.

Here, the cinnamic acids were found to be conjugated to tyramine, even though they are reported as feruloyltyramine glycoside **(22A/B)**, feruloy-3-methoxytyramine **(13A/B)** and feruloyl-3-methoxy conjugate **(15A/B),** with the glycosyl group attached either on the cinnamic acid or the tyramine. As seen in **Figure 4.13 D-E**, the molecules have the same precursor ions but different Rt, and thus the possibility of alternative annotation cannot be ignored. For example, the different retention time difference could be due to the position of the sugar, as well as *trans* or *cis* isomerization of the cinnamic acids.

References

Clifford, M. N. 1999. Chlorogenic acids and other cinnamates- nature, occurrence and dietary burden. *J. Agric. Food Chem.* 79: 362–372. doi: 10.1002/(sici)1097-0010(19990301)79:3<362::aid-jsfa256>3.0.co;2-d.

Clifford, M. N. 2000. Chlorogenic acids and other cinnamates nature, occurrence, dietary burden, absorption andmetabolism. *J. Agric. Food Chem.* 80: 1033-1042. doi: 10.1002/(sici)1097-0010(20000515)80:7<1033::aid-jsfa595>3.0.co;2-t.

Clifford, M., Johnston, K., Knigh, S., and Kuhnert, N. (2003). A hierarchical scheme for LC-MS^n identification of chlorogenic acid. *J. Agric. Food Chem*. 51, 2900–2911. doi: 10.1021/jf026187q.

Clifford, M. N., and Knight S. 2004. The cinnamoyl-amino acid conjugates of green robusta coffee beans. *Food Chem.* 87: 457–463. doi:10.1016/j.foodchem.2003.12.020.

He, W., Liu, X., Xu, H., Gong, Y., Yuan, F., and Gao, Y. 2010. On-line HPLC-ABTS screening and HPLC-DAD-MS/MS identification of free radical scavengers in Gardenia (*Gardenia jasminoides* Ellis) fruit extracts. *Food Chem.* 123: 521–528. doi:10.1016/j.foodchem.2010.04.030.

Jaiswal, R., and Kuhnert, N. 2014. Identification and characterization of the phenolic glycosides of *Lagenaria siceraria* Stand (Bottle Gourd) fruit by liquid chromatography–tandem mass spectrometry. *J. Agric. Food Chem.* 62: 1261–1271.doi.org/10.1021/jf4053989.
