# Supplementary material for: Profiling of Altered Metabolomic States in Nicotiana tabacum Cells Induced by Priming Agents
Source: Front Plant Sci. 2016 Oct 18;7:1527. doi: 10.3389/fpls.2016.01527 (PMC5068090; doi:10.3389/fpls.2016.01527)
Supplement: Supplementary Material File 3 — Characterization of phytohormone derivatives and catabolites. [file Image3.pdf]

Supplementary Material – File 3

Characterization of phytohormone derivatives and catabolites

• Profiling of altered metabolomic states in *Nicotiana tabacum* cells induced by priming agents

Msizi I. Mhlongo[1], Paul A. Steenkamp[1,2], Lizelle A. Piater[1], Ntakadzeni E. Madala[1] and Ian A. Dubery[1]*

[1]Department of Biochemistry, University of Johannesburg, Auckland Park, Johannesburg, South Africa; [2]CSIR Biosciences, Natural Products and Agroprocessing Group, Pretoria, South Africa.

*** Correspondence:**
Ian Dubery
idubery@uj.ac.za

Keywords: chlorogenic acids, defense responses, elicitors, hydroxycinnamates, tyramine, plant activators, polyamines, priming

CHARACTERISATION OF PHYTOHORMONE DERIVATIVES AND CATABOLITES

Figure S3.1. Representative extracted single ion chromatograms (XIC) of UHPLC-MS/MS showing the Rts of ABA catabolites and its derivatives: (A) at *m/z* 279.12 for 9'-hydroxy-abscisic acid, **(B)** at *m/*z 281.13 for dihydrophaseic acid, **(C)** at *m/z* 425.18 for abscisic acid glycoside and **(D)** at *m/z* 467.18 for abscisic acid conjugate and **(E)** at *m/*z 471.18 for abscisic acid glycoside conjugate.

Figure S3.2. MS spectra showing fragmentation patterns of ABA catabolites and its derivatives: (A) 9'-hydroxy-abscisic acid, **(B)** dihydrophaseic acid, **(C)** abscisic acid glycoside, **(D)** abscisic acid conjugate I and **(E)** abscisic acid conjugate II.

Figure S3.3. Single ion chromatograms of UHPLC-MS showing Rts of SA derivatives: (A) salicylic acid glycoside at *m/z* 299.07 and **(B)** methyl salicylate glycoside at *m/z* 313.08.

Figure S3.4. MS spectra showing the fragmentation pattern of SA derivatives: (A) salicylic acid glycoside and **(B) SA** methyl ester glycoside (methyl salicylate glycoside).

Figure S3.5. Single ion chromatograms of UHPLC-MS showing Rts of MJ catabolites and derivatives. (A) Jasmonic acid conjugate at *m/z* 311.16 and **(B)** at *m/z* 417.17.

Figure S3.6. MS spectra showing the fragmentation pattern of conjugated jasmonic acid (A and B).

3.1 Characterization of abscisic acid and its conjugates

In plants, ABA is metabolized by three reaction types: oxidation, conjugation, and reduction (Zeevaart and Creelman, 1988; Oritani and Kiyota, 2003). The major oxidative pathway of natural ABA [(+)-S-enantiomer] is through 8'-hydroxylation, forming 8'-hydroxy-ABA which, in turn spontaneously isomerizes to phaseic acid (PA). PA can then be reduced to the major product *di*hydrophaseic acid (DPA), with minor amounts of epi-*di*hydrophaseic acid (epi-DPA). The minor oxidation pathway includes the formation of (+)-7'-hydroxy-ABA (7-OH-ABA), while the minor reductive pathway produces the unstable ABA 1',4'-diol (Zeevaart, 1999; Oritani and Kiyota, 2003). A novel ABA 9'-hydroxylation pathway has also been reported (Zhou *et al.*, 2004), yielding the metabolites 9'-hydroxyABA (9'-OH-ABA) and its cyclized product, neophaseic acid (neoPA). In addition, ABA and its metabolites may be conjugated glycosides at C1 or C4 (Zeevaart, 1999; Oritani and Kiyota, 2003).

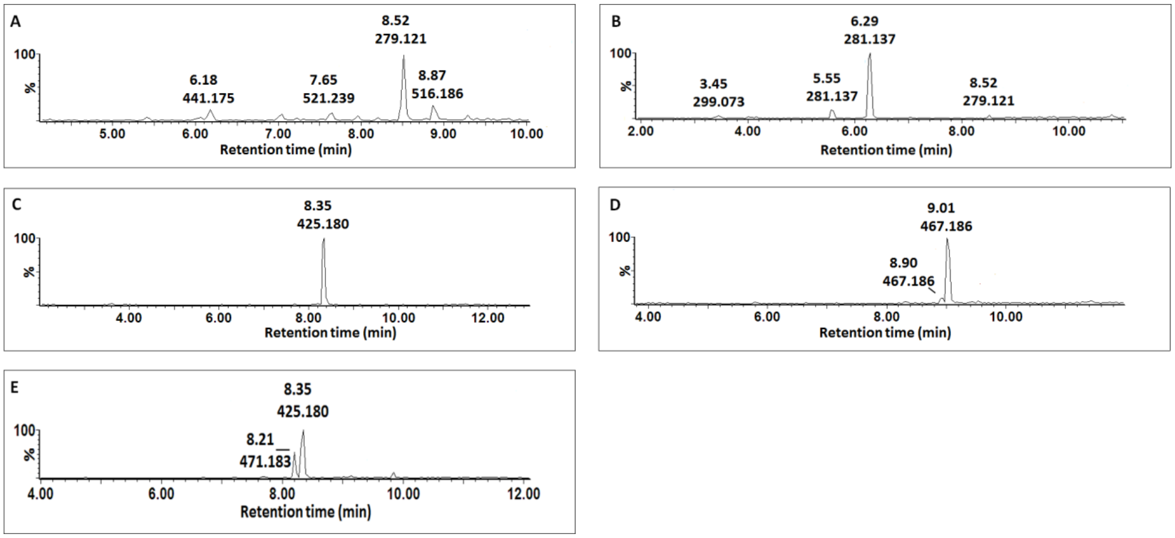

Figure S3.1: Representative extracted single ion chromatograms (XIC) of UHPLC-MS/MS showing the Rts of ABA catabolites and its derivatives: (A) at *m/z* 279.12 for 9'-hydroxy-abscisic acid, **(B)** at *m/z* 281.13 for dihydrophaseic acid, **(C)** at *m/z* 425.18 for abscisic acid glycoside and **(D)** at *m/z* 467.18 for abscisic acid conjugate and **(E)** at *m/z* 471.18 for abscisic acid glycoside conjugate.

Molecule **(20)** was annotated as *di*hydrophaseic acid with precursor ion at *m/z* 279.12 ($[M-H]^-$), Rt 6.30 min **(Fig. S3.1A)** and with a MS spectrum showing a product ion at *m/z* 205.11 **(Fig. S3.2A)**. Molecule **(26)** was annotated as 9'-hydroxy-absiscic acid with a precursor ion at *m/z* 281.18 ($[M-H]^-$), Rt 8.15 **(Fig. S3.1B)** and product ion at *m/z* 237.14 **(Fig. S3.2B)**. ABA has a molecular weight of 264.21 and its MS spectrum on negative ionization produces two product ion at *m/z* 219.03 and 153.04 (Chiwocha *et al.*, 2003; Tureckova *et al.*, 2009). Molecule **(25)** was annotated as ABA glycoside with precursor ion at *m/z* 425.18 ($[M-H]^-$), Rt 8.35 (**Fig. S3.1C).** Its MS spectrum show a product ion at 263.12 ($[M-H-162\ Da]^-$) resulting from a glycosyl residue loss and two product ions at *m/z* 219.13 and 153.09 corresponding ABA product ions **(Fig. S3.2C)**. Molecule **(27)** was annotated as ABA conjugate II with a precursor ion at *m/z* 467.18 ($[M-H]^-$), Rt 9.02 min **(Fig. S3.1D)** and with a MS spectrum showing product ions at *m/z* 219.11 (with loss of 204 Da which could not be

assigned) and at *m/z* 153.09, which are ABA fragments **(Fig. S3.2D)**. Molecule **(24)** was annotated as ABA conjugate I with a precursor ion at *m/z* 471.18 ([M-H]$^-$), Rt 8.28 min **(Fig. S3.1E)** and with a MS spectrum showing product ions at 263 ([M-208 Da-H$^-$]), *m/z* 219.11 (with loss of 208 Da which could not be assigned) and at *m/z* 153.09, which are ABA fragments **(Fig. S3.2E)**. The accumulation of both ABA glycosides, catabolites and ABA conjugates could be a possible mechanism employed by plant to detoxify, store or transport ABA.

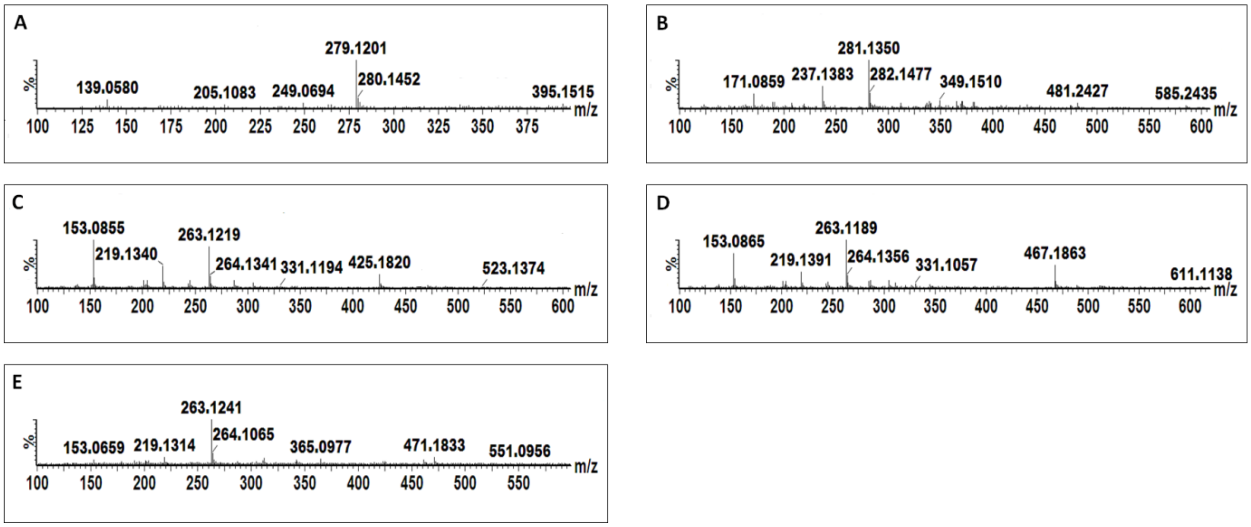

Figure S3.2. MS spectra showing fragmentation patterns of ABA catabolites and its derivatives: (A) 9'-hydroxy-abscisic acid, **(B)** dihydrophaseic acid, **(C)** abscisic acid glycoside, **(D)** abscisic acid conjugate I and **(E)** abscisic acid conjugate II.

3.2 Characterization of salicylic acid glycosides and methylsalicylate glycoside

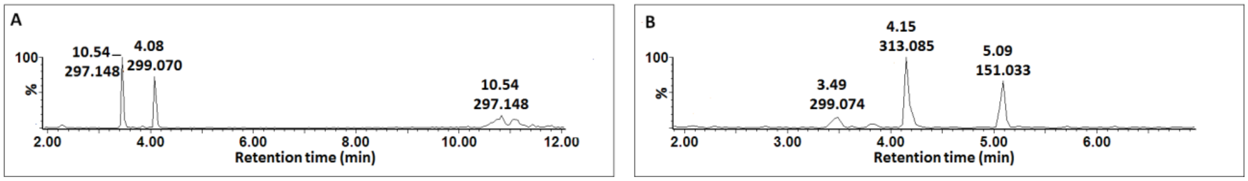

Figure S3.3. Single ion chromatograms of UHPLC-MS showing Rts of SA derivatives: (A) salicylic acid glycoside at *m/z* 299.07 and **(B)** methyl salicylate glycoside at *m/z* 313.08.

Molecule **(17)** was annotated as SA glycoside with a precursor ion at *m/z* 299.07 ($[M-H]^-$), Rt 4.04 min **(Fig. S3.3A)** and its MS spectrum showed a product ion at *m/z* 137.01 ($[M-H-162\ Da]^-$) indicating a glycosyl residue loss **(Fig. S3.4A)**. Molecule **(18)** was annotated as MeSA glycoside with a precursor ion at *m/z* 313 ($[M-H]^-$), Rt 4.15 (**Fig. S3.3B)** and its MS spectrum showing a product ion at *m/z* 151.05 ($[M-H-162\ Da]^-$) after the elimination of a glycosyl residue **(Fig. 3.4B)**

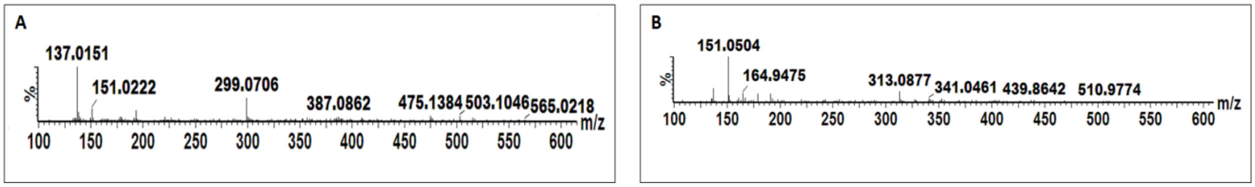

Figure S3.4. MS spectra showing the fragmentation pattern of SA derivatives: (A) salicylic acid glycoside and **(B) SA** methyl ester glycoside (methyl salicylate glycoside).

As reported elsewhere, the detection of SA glycoside in ASM-treated cells indicates that ASM uses the SA pathway to induce a primed state in tobacco cells. Priming by pathogens is associated with high concentrations of SA and MeSA. Here, the accumulation of both SA glycoside and MeSA glycoside indicates that the pathogen-derived elicitors induce a primed state by stimulating the SA signaling pathway. The accumulation of SA glycoside and MeSA glycoside in SA-treated cells can also be explained in the context of storage-, transportation- and detoxification mechanisms in tobacco cells.

3.3 Characterization of jasmonic acid conjugates

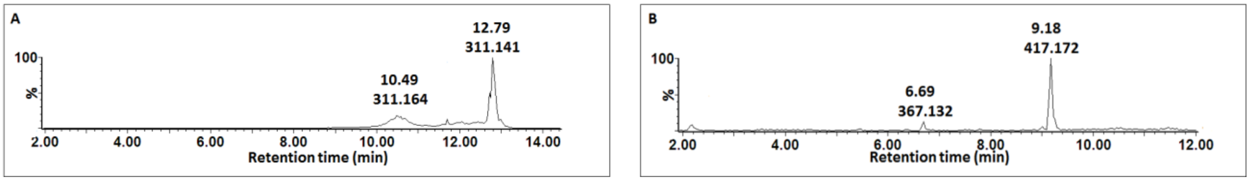

Figure S3.5. Single ion chromatograms of UHPLC-MS showing Rts of MJ catabolites and derivatives. (A) Jasmonic acid conjugate at *m/z* 311.16 and **(B)** at *m/z* 417.17.

Molecules **(28)** and **(29)** were annotated as JA conjugates with product ions at *m/z* 311.16 ($[M-H]^-$), Rt 10.39 min **(Fig. 3.5A)** and *m/z* 417.17 ($[M-H]^-$), Rt 9.12 min respectively **(Fig. 3.5A).** Both their MS spectra showed a product ion at *m/*z 209.11 11 (after a loss of molecules with molecular weights of 102 Da and 208 Da, respectively) which correspond to ($[JA-H]^-$) **(Fig. 3.6A and Fig. 3.6 B)**. Accumulation of these two conjugates could be a way for the cells to detoxify, store or transport JA and it methyl ester, MJ.

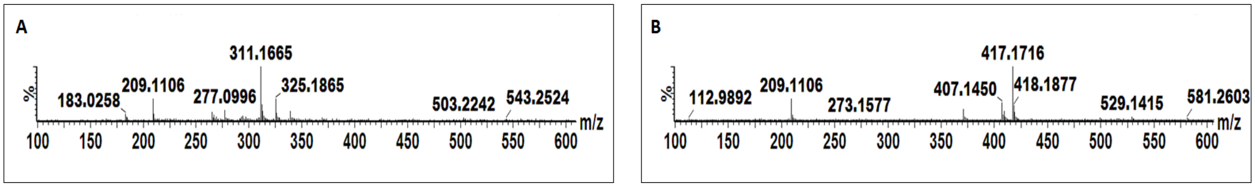

Figure S3.6. MS spectra showing the fragmentation pattern of conjugated jasmonic acid (A and B).

References

Chiwocha, S. D. S., Abrams, S. R., Ambrose, S. J., Cutler, A. J., Loewen, M., Ross, A. R. S., and Kermode A. R. 2003. A method for profiling classes of plant hormones and their metabolites using liquid chromatography-electrospray ionization tandem mass spectrometry : an analysis of hormone regulation of thermodormancy of lettuce (*Lactuca sativa* L.) seeds. *Plant J.* 35: 405–417. doi: 10.1046/j.1365-313X.2003.01800.x.

Oritani, T., and Kiyota, H. 2003. Biosynthesis and metabolism of abscisic acid and related compounds. *Nat. Prod. Rep* 20: 414–425. doi: 10.1039/b109859b.

Tureckova, V., Novak, O., and Strnad, M. 2009. Profiling *Nicotiana tabacum* L. leaves by ultra-performance liquid chromatography – electrospray tandem mass spectrometry. *Talanta* 80: 390-399. doi:10.1016/j.talanta.2009.06.027.

Zeevaart, J. A. D., and Creelman, R. A. 1988. Metabolism and physiology of abscisic-acid. *Ann. Rev Plant Physiol. Plant Mol. Biol.* 39: 439–473. doi: 10.1146/annurev.pp.39.060188.002255.
